# P2X7 is an important mediator of BMP9-induced osteogenic differentiation of mesenchymal stem cells

**DOI:** 10.1186/s12964-026-02747-w

**Published:** 2026-02-25

**Authors:** Lulu Zhang, Ziyun Li, Yannian Gou, Habu Jiwa, Jiayu Wang, Wei Zhang, Jinyong Luo

**Affiliations:** 1 Department of Clinical Laboratory, The University-Town Hospital of Chongqing Medical University, Chongqing, 401331 China; 2 Medical Sciences Research Center, The University-Town Hospital of Chongqing Medical University, Chongqing, 401331 China; 3 Key Laboratory of Laboratory Medical Diagnostics, Ministry of Education, Chongqing Medical University, No.1 Yixueyuan Road, Yuzhong District, 400016 Chongqing, P.R. China; 4 Department of Orthopedics, The First Affiliated Hospital of Chongqing Medical University, Chongqing, 400016 China; 5 Department of Clinical Laboratory, Peking University Shenzhen Hospital, Shenzhen, 518036 China

**Keywords:** BMP9, P2X7, Osteogenic differentiation, Mesenchymal stem cells

## Abstract

**Background:**

Bone morphogenetic protein 9 (BMP9) has been demonstrated to robustly induce osteogenic differentiation of mesenchymal stem cells (MSCs), offering substantial potential for advancements in bone tissue engineering. The purinergic receptor P2X7 has emerged as a crucial modulator of bone formation and bone metabolism. However, the precise role of P2X7 in BMP9-induced osteogenic differentiation of MSCs and the associated molecular mechanisms remain partially understood. This study aims to ascertain the exact function of P2X7 in BMP9-induced osteogenic differentiation of MSCs, and to unravel the relevant molecular mechanism.

**Methods:**

Transcriptome sequencing, quantitative real-time polymerase chain reaction, Western blot, and chromatin immunoprecipitation assays were initially performed to validate the up-regulation of P2X7 by BMP9. Subsequently, the influence of P2X7 on BMP9-induced osteogenic differentiation of MSCs was assessed through ALP determination, calcium deposition analysis, Western blotting, ectopic bone formation model, and skull defect model. Finally, the mechanism through which P2X7 modulated BMP9-induced osteogenic differentiation of MSCs was investigated using intracellular Ca^2+^ imaging, Western blot, molecular docking, immunoprecipitation and immunofluorescence staining.

**Results:**

BMP9 was confirmed to upregulate P2X7 expression via Smad signaling. Activation of P2X7 significantly potentiated BMP9-induced osteogenic differentiation of MSCs and enhanced BMP9-promoted ectopic bone formation, then enhanced the repair of bone defect by BMP9. Conversely, inhibition of P2X7 elicited a contrary effect. Mechanistically, P2X7 activation promoted calcium influx, subsequently leading to the activation of CaMKII by phosphorylation. The activated CaMKII then interacted with GSK-3β, facilitating the inhibitory phosphorylation of GSK-3β at Serine 9 (Ser 9) residue. This may stabilize β-catenin and increase its nuclear translocation, thus finally mediating the osteogenic differentiation of MSCs induced by BMP9.

**Conclusion:**

This study clarifies that P2X7 may mediate BMP9-induced osteogenic differentiation of MSCs through the CaMKII/GSK-3β/β-catenin axis, providing novel insights into the molecular mechanism of BMP9-induced osteogenesis and a potential target for bone defect treatment.

**Supplementary Information:**

The online version contains supplementary material available at 10.1186/s12964-026-02747-w.

## Background

Bone defects caused by physiological and pathological factors, including but not limited to trauma, infection, tumors or functional atrophy, pose a significant challenge in clinical practice. In the event of minor bone damage, the body is generally capable of self-repair and regeneration [1]. When there is a large defect, it needs to be repaired with the help of external forces. Conventional treatment strategies, including autogenous and allogeneic bone transplantation, are inherently limited by their source and are prone to complications or immune rejection [2, 3]. In recent years, however, the advent of bone tissue engineering presents a promising alternative for addressing bone defects [4, 5].

Mesenchymal stem cells (MSCs) are a type of cell characterized by their multidirectional differentiation potential, strong tissue repairing capacity, low immunogenicity and minimal ethical concerns [6]. These attributes render them ideal candidates for bone tissue engineering. Simultaneously, the differentiation of MSCs towards osteoblastic lineage is inseparable from the directional osteo-inductive action of osteogenic factors, which exert a predominant influence on the quality of MSCs-based new bone formation [7]. The family of bone morphogenetic proteins (BMPs), initially purified from vertebrate bone matrix, possesses multiple biological functions and is essential for the development, repair and regeneration of the entire skeletal system [7, 8]. Several members of the BMPs family have been clearly validated to effectively induce osteogenic differentiation of MSCs both in vitro and in vivo. Of these, BMP9 is considered to be the most robust [9]. In regulating the process of osteogenesis, BMP9 mainly transmits signals through two receptors and SMAD molecules. BMP9 binds to type II receptors and triggers phosphorylation of type I receptors, thereby activating downstream Smad proteins. This will lead to the translocation of Smad to the nucleus, where they act as transcription factors to regulate osteogenic responses [10]. Besides directly activating the canonical Smads pathway, BMP9 also orchestrates the osteogenic differentiation of MSCs through non-canonical Smad-independent pathways, including Notch, Wnt/β-catenin and PKA/CREB signaling [11–14]. Although these advances emphasize the crucial role of BMP9 in osteogenesis, the molecular mechanism of its action is still not fully elucidated. Consequently, systematic exploration of the mechanism of BMP9 in MSCs bone induction has profound guiding significance for the development of bone tissue engineering and clinical bone regeneration of MSCs.

The purinergic receptor P2X7 (also known as P2RX7 or P2X7R) is an ATP-gated non-selective cation channel belonging to the P2X receptor family, which comprises seven receptor isoforms encoded by the P2X1-P2X7 genes [15, 16]. Upon ATP binding to the extracellular “pocket” of P2X7, it triggers a sequence of events including Ca²⁺ and Na⁺ influx, and K⁺ outflow [17]. P2X7 is widely expressed in osteoblasts, and its absence in mice leads to a significant reduction in the number of osteoblasts [18]. Activation of P2X7 in osteoblasts may promote their proliferation, differentiation and maturation, thereby accelerating bone formation [19]. P2X7 also exerts a significant regulatory effect on the osteogenic differentiation of MSCs. For instance, increased P2X7 expression is observed during the osteogenic differentiation process of adipose-derived MSCs, and its inhibition results in attenuated osteogenic differentiation [20]. Activation of P2X7 in bone marrow mesenchymal stem cells (BMSCs) from postmenopausal women further inhibits Rho-kinase activity and promotes osteogenic differentiation and mineralization of BMSCs, suggesting its potential as a therapeutic target for postmenopausal bone loss [21]. Here, we highlight that P2X7 is an essential mediator of BMP9-induced osteogenic differentiation of MSCs, potentially operating through the CaMKII/GSK-3β/β-catenin axis.

## Methods

### Cell culture

C2C12 and C3H10T1/2 cells were obtained from the American Type Culture Collection (ATCC). The cells were cultivated in Dulbecco’s modified eagle medium (DMEM, Samike, China) with the supplementation of 10% fetal bovine serum (FBS) (WISENTING, China for C2C12; Cellmax, China for C3H10T1/2) at 37 °C with 5% CO_2_.

### Isolation of BMSCs

Four-week-old male Sprague-Dawley (SD) rats (for rBMSCs) and C57BL/6 mice (for mBMSCs) were used. Femora and tibiae were harvested, and residual soft tissues were carefully removed. Both ends of the bones were cut open, and the marrow cavity was flushed with PBS until it appeared white. Collect cells by centrifugation, resuspend them in DMEM medium with 10%FBS. hBMSCs were collected from discarded bone marrow fluid of patients undergoing hip replacement in clinical practice. Cells were then seeded into T75 flasks and cultured under 37 °C with 5% CO_2_. BMSCs at passages 2 to 5 were used for subsequent experiments.

### Next-generation RNA-sequencing analysis

BMSCs were infected with adenovirus expressing BMP9 (Ad-BMP9) or Ad-GFP as a negative control for 48 h. Total RNA was extracted using TRIZOL reagent (Invitrogen, China) and assessed for integrity with the Bioanalyzer 2100 system (Agilent Technologies, CA, USA). Subsequently, sequencing was performed on the Illumina Hiseq platform for 150 bp-end reads. Sequenced reads were aligned to the reference genome using Hisat2 v2.0.5. The data processing was undertaken using Feature Counts v1.5.0-p3. Principal component analysis (PCA) was performed using the FactoMineR and factoextra software packages, and the results were visualized by the pca3d software package. The differential expression analysis was conducted using the edgeR and Limma software packages. Genes with a |log2FC| value > 1, as determined by DESeq2, were designated as altered expression genes. Genes with an adjusted P value (FDR) < 0.05 were classified as differentially expressed genes (DEGs). The KOBAS 3.0 program was utilized to perform KEGG analysis on the DEG, with the top 20 results of the classical signaling pathway subsequently being visualized.

### Quantitative Real-Time Polymerase Chain Reaction (qRT-PCR) analysis

High-quality total RNA was extracted using an RNA Extraction Kit (Accurate Biotechnology, China). According to the manufacturer’s instructions, 2 µg of total RNA was reverse-transcribed into cDNA by the Reverse Transcription Kit (MCE, USA). Next, qRT-PCR was performed using the SYBR Green qPCR Master Mix (Bimake, USA). The relative gene expression was calculated by means of the 2^(-ΔΔCT) method. The sequence of primers (TSINGKE, China) used was listed in Supplementary Table 1. GAPDH was employed as the internal reference for normalization.

### Intracellular Ca^2+^ measurement

The cells were spread on a 25 mm climbing plate. After 24 h of predetermined treatment (5mM BzATP, 1µM A740003 for C2C12; 1mM BzATP and 1µM A740003 for C3H10T1/2. The transfection efficiency of Ad-BMP9, Ad-P2X7, Ad-si1, and Ad-si2 was determined as approximately 80% based on fluorescence-positive cells 24 h post-viral addition. ), the culture medium was removed, and the cells were washed three times with PBS. Next, the cells were incubated with the Flou-4 AM working solution for 60 min, and then washed three times with PBS. The cells were then incubated with 4’,6-diamidino-2-phenylindole (DAPI) (Solarbio, Beijing, China) for 5 min to visualize the nucleus. Fluorescence images were captured using a fluorescence microscope (DMi8, Leica, Germany).

### Western blot

The cells were lysed on ice for 30 min with RIPA lysis buffer containing 1% protease/phosphatase inhibitors (Roche, Basel, Switzerland). After centrifugation, the protein concentration of cell lysate was determined using a BCA Protein Assay Kit (Beyotime, China). The protein samples were separated by SDS-PAGE, and then transferred onto a polyvinylidene fluoride membrane (Millipore, USA). After blocking with 5% Bovine Albumin V (Solarbio, Beijing, China), the membrane was subjected to overnight incubation with primary antibodies at 4 °C, and subsequently with horseradish peroxidase (HRP)-conjugated secondary antibodies (ZSGB-BIO, China) at room temperature for 1 h. Visualization of the target protein was achieved using an ECL kit, with images captured using the gel imaging system. Details of the primary antibodies were provided in Supplementary Table 2.

### Alkaline Phosphatase (ALP) staining

Cells were implanted into a 24 well plate. C2C12 cells were subjected to different treatments (BzATP: 5mM, A740003:1µM, KN93: 3µM) for 5 days. C3H10T1/2 cells were subjected to different treatments (BzATP: 1mM, A740003: 1µM, KN93: 5µM) for 7 days. Primary BMSCs were subjected to different treatments for 7 days. The transfection efficiency of Ad-BMP9, Ad-P2X7, Ad-si1, and Ad-si2 was determined as approximately 80% based on fluorescence-positive cells 24 h post-viral addition. After treatments, the cells were fixed for 20 min with 4% paraformaldehyde, followed by a thorough washing process involving two cycles of PBS. Subsequently, the cells were stained with 250 µL of BCIP/NBT substrate (Solarbio, Beijing, China) for 20 min in a dark environment. The ALP staining results were then captured using an optical microscope. (BzATP, MCE, America; A740003, Selleck, America; KN93, MCE, America).

### Alkaline phosphatase activity

Cells were implanted into a 24 well plate. C2C12 cells were subjected to different treatments (BzATP: 5mM, A740003:1µM, KN93: 3µM) for 5 days. C3H10T1/2 cells were subjected to different treatments (BzATP: 1mM, A740003: 1µM, KN93: 5µM) for 7 days. The transfection efficiency of Ad-BMP9, Ad-P2X7, Ad-si1, and Ad-si2 was determined as approximately 80% based on fluorescence-positive cells 24 h post-viral addition. After treatment, the culture medium was discarded, and then 100 µL of cell lysis buffer was added to each well to completely lyse all cells. Subsequently, cell lysates were transferred to Eppendorf tubes and subjected to centrifugation (4500×g, 5 min). Next, 5µL of the supernatant was mixed with pre-configured working solution comprising 5 µL of ALP substrate and 15 µL of buffer. After 45–60 min of reaction in the dark, ALP activity was measured quantitatively using an automatic biochemical analyzer.

### Alizarin red staining

Cells were implanted into a 24 well plate. C2C12 cells were subjected to different treatments (BzATP: 5mM, A740003:1µM, KN93: 3µM) for 5 days. C3H10T1/2 cells were subjected to different treatments (BzATP: 1mM, A740003: 1µM, KN93: 5µM) for 7 days. The transfection efficiency of Ad-BMP9, Ad-P2X7, Ad-si1, and Ad-si2 was determined as approximately 80% based on fluorescence-positive cells 24 h post-viral addition. During this period, the cells were continued to be maintained in osteogenic induction medium (20 mM β-glycerophosphate, 50 µM L-ascorbic acid-2-phosphate, and 2% FBS in DMEM; Samike, China). At designated time intervals, the media were discarded, and the cells were rinsed with PBS. Mineral deposition was subsequently visualized by stained with 250 µL of 2% alizarin red S staining solution.

### Ectopic bone formation model

C3H10T1/2 cells were cultured in 10 cm dishes and received various treatments according to the pre-grouping (Control, BMP9, BMP9 + Ad-P2X7, BMP9 + Ad-si1, and BMP9 + Ad-si2, *n* = 5). The transfection efficiency of Ad-BMP9, Ad-P2X7, Ad-si1, and Ad-si2 was determined as approximately 80% based on fluorescence-positive cells 24 h post-viral addition. After 24-hours of treatment, cells were harvested, resuspended in PBS, and injected subcutaneously into the lateral back of thymus-free nude mice (5-week-old females from Jiangsu Biotechnology Co., Ltd.), at a dose of 5 × 10⁶ cells per injection (20 µL PBS). After 30 days of injection, the animals were euthanized and the subcutaneous masses formed were harvested for micro-computed tomography (micro-CT) analysis (SCANCO Medical, Bruttisellen, Switzerland) and histological staining analysis.

### Calvarial defect model

rBMSCs were isolated and cultured in 10 cm dishes, and then received different treatments. The transfection efficiency of Ad-BMP9, Ad-P2X7, Ad-si1, and Ad-si2 was determined as approximately 80% based on fluorescence-positive cells 24 h post-viral addition. After 24 h, the cells were collected (3 × 10^6^ cells) and mixed with 10 µL 5% Gelatin methacryloyl (GelMA/GM). Next, 5-week-old male SD rats were anesthetized and dissected to expose their skulls. After creating a defect of 5 mm diameter on the right side of the skull, and the animals were randomly grouped (Control, GM, GM+BMSCs, GM+BMP9, GM+BMP9 + Ad-P2X7, GM+BMP9 + Ad-si1, GM+BMP9 + Ad-si2, *n* = 5) [22]. Next, the GM or GM/BMSCs mixture was injected into each defect according to the grouping, followed by 10 s of exposure to ultraviolet light. After 45 days of calvarial defect establishment, the animals were euthanized and their calvarial bones were collected for micro-CT imaging and histological staining.

### H&E staining, Masson staining and Alcian blue staining

The retrieved samples of each group were processed into paraffin blocks. Then, 5 μm thick sections were prepared from paraffin-embedded samples, followed by rehydration using 100%, 90%, 80%, and 70% ethanol sequentially. Sections were then stained with Hematoxylin-Eosin (Solarbio, Beijing, China), Masson’s trichrome staining (Masson’s trichrome staining kit, Sigma) and Alcian Blue staining (Solarbio, Beijing, China) according to the standard histopathological protocol. The images of different staining assays were photographed under a light microscope.

### MTT assay

rBMSCs were seeded in 96-well plates at a density of 10⁴ cells/well and allowed to attach. The rBMSCs were then treated with 10 µL of 5% GelMA. At the indicated time point, 200 µL of 5 mg/mL MTT solution (Solarbio, Beijing, China) was added and incubated at 37 °C for another 4 h. The MTT solution was then removed, and 50 µL of DMSO (BioFROXX, Germany) was added to each well to ensure complete dissolution of the MTT formazan. The assessment of cell viability was conducted by quantifying the optical density at a wavelength of 492 nm using a multifunctional enzyme labeling detector.

### Chromatin Immunoprecipitation (ChIP)

The ChIP assay was carried out using the BeyoChIP™ ChIP Assay Kit (Beyotime, China) following the manufacturer’s standard protocol. Briefly, C3H10T1/2 cells were cultivated adherently on 10 cm dishes and subjected to Ad-BMP9 or Ad-GFP treatments. After 48 h, cells were crosslinked with 1% formaldehyde in culture medium for 10 min, followed by treatment with 11.1 mL of Glycine Solution (10×) for 5 min. Following a thorough washing step with PBS containing PMSF at a final concentration of 1 mM, the cells were mixed with 0.2 mL of SDS Lysis buffer and left on ice for 10 min. The cells were sonicated (130 W, 37% power) for 80 s to break the genomic DNA, and then were centrifuged (14,000×g) at 4 °C for 5 min. Next, the supernatant was transferred to a new Eppendorf tube, and mixed with 1.8 mL of Chip Dilution Buffer. After taking 20 µL as input, the remaining samples were mixed with 70 µL of Protein A/G for 30 min at 4℃ (slowly rotate). After being centrifuged, the supernatant was incubated with 1 µg of anti-Smad4 or anti-Smad5 or pre-immune IgG (Protentech, China) overnight at 4 °C with shaking. Subsequently, 60 µL of Protein A/G was added and the mixture was continued to incubate at 4 °C for 4 h. After centrifugation, the residual precipitate was dissolved in TE buffer and the DNA was purified using a MinElute PCR Purification Kit (Beyotime, China). Subsequent to this, the purified DNA was quantitatively analyzed by PCR.

### Immunoprecipitation

The cells were lysed with RIPA lysis buffer on ice for 30 min, followed by centrifugation at 13,000×g for 15 min to collect cell lysates. Thereafter, antibodies were added to the supernatant and incubated at 4 °C overnight. Next, protein A/G beads were added and allowed to react for another 2 h. After being centrifugated (13000×g, 15 min), the protein A/G beads were washed with lysis buffer (50 mM Tris + 150 mM NaCl + 0.5% Tween), and subjected to Western blot analysis.

### Immunofluorescence staining

The cells were fixed with 4% paraformaldehyde, and then permeabilized for 6 min with 0.5% Triton X-100 (Solarbio, Beijing, China). Then, the cells were blocked with 5% BSA for 1 h, followed by incubation with primary antibodies overnight at 4 °C. Subsequently, the cells were washed thrice with PBS and incubated with conjugated secondary antibody for 1 h. After a five-minute incubation with DAPI, the fluorescence signal was captured using a fluorescence microscope (DMi8, Leica, Germany).

### Live/Dead assays

rBMSCs were seeded into 96-well plates at a density of 10⁴ cells per well and allowed to adhere. Subsequently, the cells were mixed with 10 µL of 5% GelMA hydrogel, followed by crosslinking using ultraviolet (UV) light for 8–10 s. Thereafter, the cells were cultivated in complete DMEM medium for 1, 3, 5, and 7 days, respectively. After that, the cells underwent triple washing with PBS, and were incubated with 100 µL of working solution (1×Assay Buffer: Calcein-AM: PI = 1000:1:3) for 15 min at 37 °C. The distinction between live (green) and dead (red) cells was visualized through fluorescence microscopy.

### Statistical analysis

The data in this study were from at least three independent analyses. All quantitative data were presented as mean ± SEM, and were analyzed using GraphPad Prism 8.0 (GraphPad Software, La Jolla, CA). Statistical analysis between the two groups was conducted using two-tailed Student’s t-tests, and the mutual comparison among multiple groups was determined by one-way analysis of variance (ANOVA) Tukey’s test, with a significance level set at *P* < 0.05.

## Results

### BMP9 upregulates P2X7 expression through Smad signaling

**Fig. 1:**
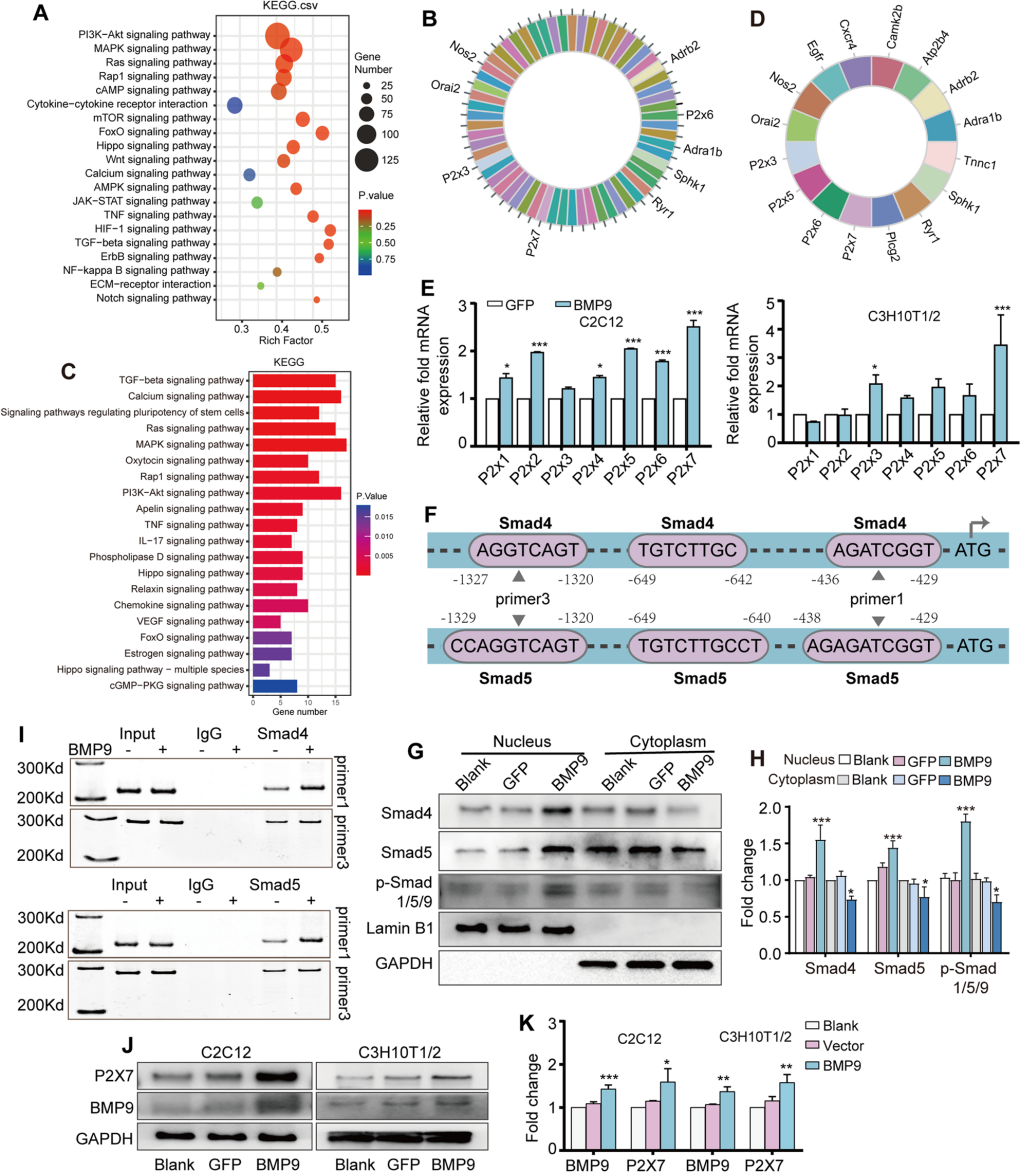
BMP9 promotes P2X7 expression via Smad signaling in MSCs. **A** KEGG enrichment analysis to depict the significantly changed signaling pathway in C3H10T1/2 cells after BMP9 treatment. **B** Enriched genes in the calcium signaling pathway. **C** KEGG results of the significantly changed signaling pathways in BMSCs after BMP9 treatment. **D** Visualization of the genes enriched in calcium signaling pathway. **E** qRT-PCR to detect the mRNA levels of P2X family in MSCs after BMP9 treatment. **F** Schematic diagram of the binding site of Smad4 and Smad5 on the P2X7 promoter region. **G** Western blot assay to detect the distribution of Smad4, Smad5 and p-Smad 1/5/9 in the nucleus and cytoplasm. **H** Quantitative analysis of the Western blot results in Fig. 1E. **I** ChIP assay to validate the binding of Smad4 and Smad5 to P2X7 promoter region. **J** Western blot to detect the protein level of P2X7 in MSCs after BMP9 treatment. **K** Quantitative analysis of Western blot results in Fig. 1H. *P < 0.05; **P < 0.01; ***P < 0.001

Based on the RNA sequencing results, we observed that among the top 20 KEGG pathways enriched in BMP9 stimulated C3H10T1/2 and BMSCs, the calcium signaling pathway ranked high, which has attracted our special attention due to its importance in bone development and MSC osteogenic differentiation (Fig. 1A and C) [23, 24]. Analysis of genes involved in the calcium signaling pathway highlighted enrichment of multiple P2X family members, particularly P2X3, P2X6, and P2X7 (Fig. 1B and D), with P2X7 exhibiting the most pronounced transcriptional response to BMP9 (Fig. 1E and Fig.S1A). Further analysis of the P2X7 promoter sequence using JASPAR identified three conserved Smad4/5 binding sites within the P2X7 promoter region (Fig. 1F). Accordingly, the levels of Smad4, Smad5 and p-Smad 1/5/9 proteins in the nucleus were found to dramatically increase after BMP9 treatment (Fig. 1G and H), suggesting the activation of Smad signaling. Next, the ChIP experiment confirmed the enriched presence of Smad4 and Smad5 on the P2X7 promoter region following BMP9 treatment (Fig. 1I). Additionally, BMP9 treatment led to increased levels of P2X7 protein in MSCs as well (Fig. 1J and K). Interestingly, we found that BMP2 and BMP4 can also promote the mRNA and protein expression of P2X7, indicating that P2X7 is not specific to BMP9 and may be regulated by other members of the BMPs family (Fig.S2A-C). In conclusion, these results establish P2X7 as a direct target of BMP9-induced Smad signaling.

### P2X7 regulates BMP9-induced osteogenic differentiation of MSCs in vitro and in vivo

**Fig. 2:**
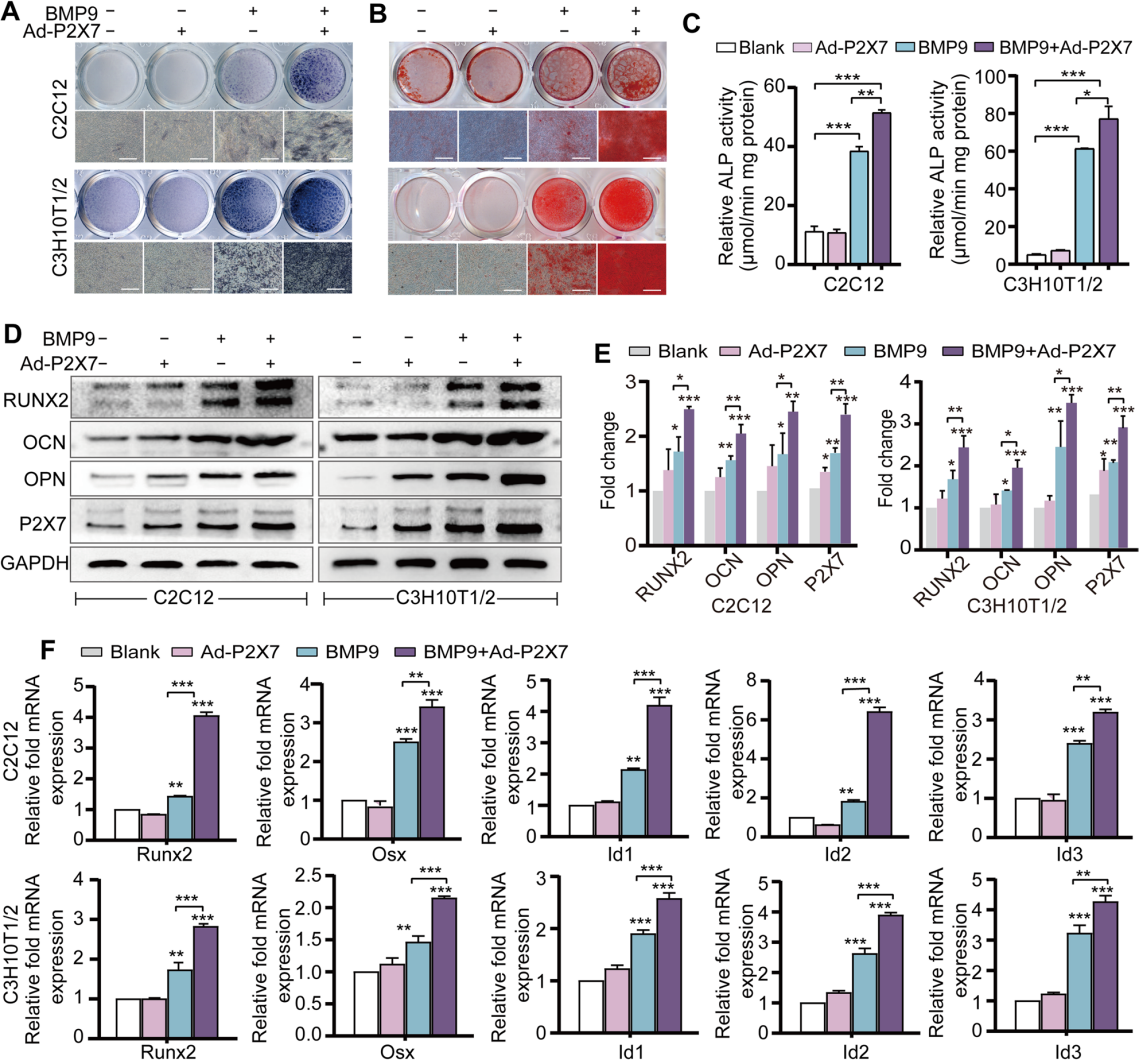
Overexpression of P2X7 by adenovirus Ad-P2X7 enhances BMP9-induced osteogenic differentiation in MSCs. **A** ALP staining to detect the early osteogenic ability of MSCs after BMP9 treatment in the presence of Ad-P2X7. Scale bars, 100 μm. **B** Alizarin red staining to assess the calcium deposition of MSCs after BMP9 treatment in the presence of Ad-P2X7. Scale bars, 100 μm. **C** ALP activity quantification assay to assess the early osteogenic ability of MSCs after BMP9 treatment in the presence of Ad-P2X7. **D** Western blot to detect the effect of P2X7 overexpression on the protein levels of osteogenic markers. **E** Quantitative analysis of Western blot results in Fig. 2D. **F** qRT-PCR to analyze the effect of P2X7 overexpression on the mRNA levels of osteogenic markers. *P < 0.05; **P < 0.01; ***P < 0.001

**Fig. 3:**
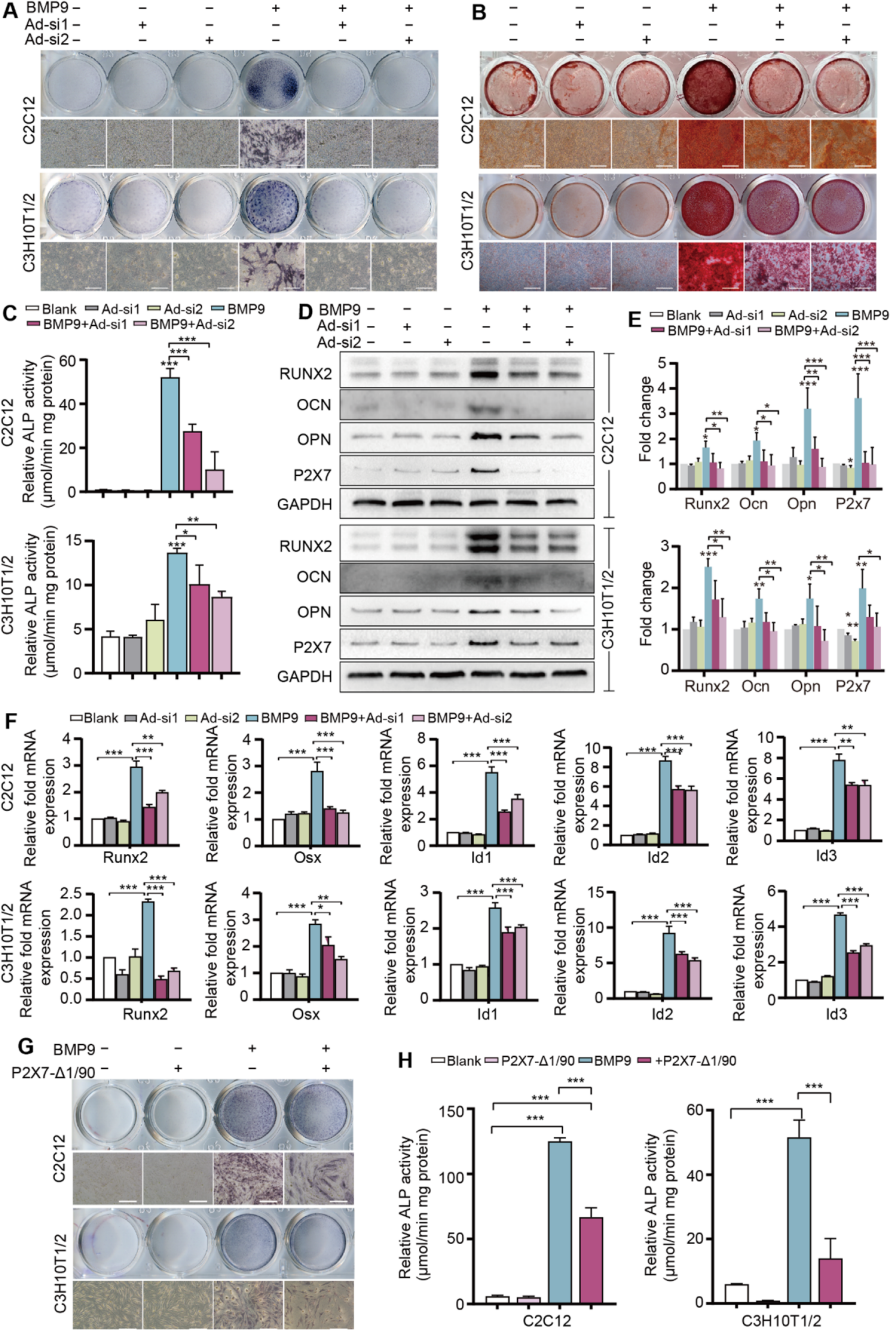
Interference of P2X7 expression or loss-of-function of P2X7 inhibits BMP9-induced osteogenic differentiation of MSCs.** A** ALP staining to analyze the effect of P2X7 silencing on BMP9-driven early osteogenic differentiation of MSCs. Scale bars, 100 μm. **B** Alizarin red S staining to assess the effect of P2X7 silencing on BMP9-driven late osteogenic differentiation of MSCs. Scale bars, 100 μm. **C** ALP activity quantification to assess the effect of P2X7 silencing on BMP9-driven early osteogenic differentiation of MSCs. **D** Western blot to detect the protein levels of osteogenic markers in BMP9-treated MSCs in the presence of Ad-si1 and Ad-si2. **E** Quantitative analysis of Western blot results in Fig. 3D. **F** qRT-PCR to measure the mRNA levels of osteogenic markers in BMP9-treated MSCs in the presence of Ad-si1 and Ad-si2. **G** ALP staining to assess the early osteogenic differentiation of BMP9-treated MSCs in the presence P2X7 lost-of-function mutation (P2X7-Δ1/90). **H** ALP activity quantification to evaluate the early osteogenic differentiation of BMP9-treated MSCs in the presence P2X7-Δ1/90. Scale bars, 100 μm. *P < 0.05; **P < 0.01; ***P < 0.001

Further investigation was conducted to determine the functional contribution of P2X7 to BMP9-driven osteogenic differentiation of MSCs. The pharmacological activation of P2X7 with BzATP markedly enhanced BMP9-induced ALP activity and calcium deposition in MSCs, whereas the P2X7 antagonist A740003 significantly attenuated this effect of BMP9 on MSCs (Fig.S3A-C). In addition, BzATP significantly increased the mRNA levels of typical osteogenic factors up-regulated by BMP9, while A740003 inhibited them (Fig.S3D). Subsequently, we validated the potency of adenovirus overexpressing P2X7 (Ad-P2X7), and adenovirus expressing small interfering RNA for P2X7 (Ad-si1 and Ad-si2) (Fig.S4A-F). As anticipated, Ad-P2X7 further potentiated early and late osteogenic differentiation in BMP9-treated MSCs, leading to higher ALP activity and calcium deposition (Fig. 2A-C). Furthermore, compared to BMP9 alone, the protein and mRNA levels of osteogenesis-related molecules were also higher when Ad-P2X7 was combined with BMP9 (Fig. 2D-F). In contrast, Ad-si1 and Ad-si2 impaired the early and late osteogenic differentiation of MSCs promoted by BMP9, as well as the proteins and mRNA levels of osteogenic molecules (Fig. 3A-F). Additionally, P2X7-Δ1/90, a truncated loss-of-function mutant of P2X7 [25], also attenuated the osteogenic induction function of BMP9 in MSCs (Fig. 3G and H). Then we applied the corresponding treatment to mBMSCs and found that the results were similar to those observed in C2C12 and C3H10T1/2 (Fig.S1B and C). It appears that P2X7 may potentially influence BMP9-induced osteogenic differentiation of MSCs in vitro.

**Fig. 4:**
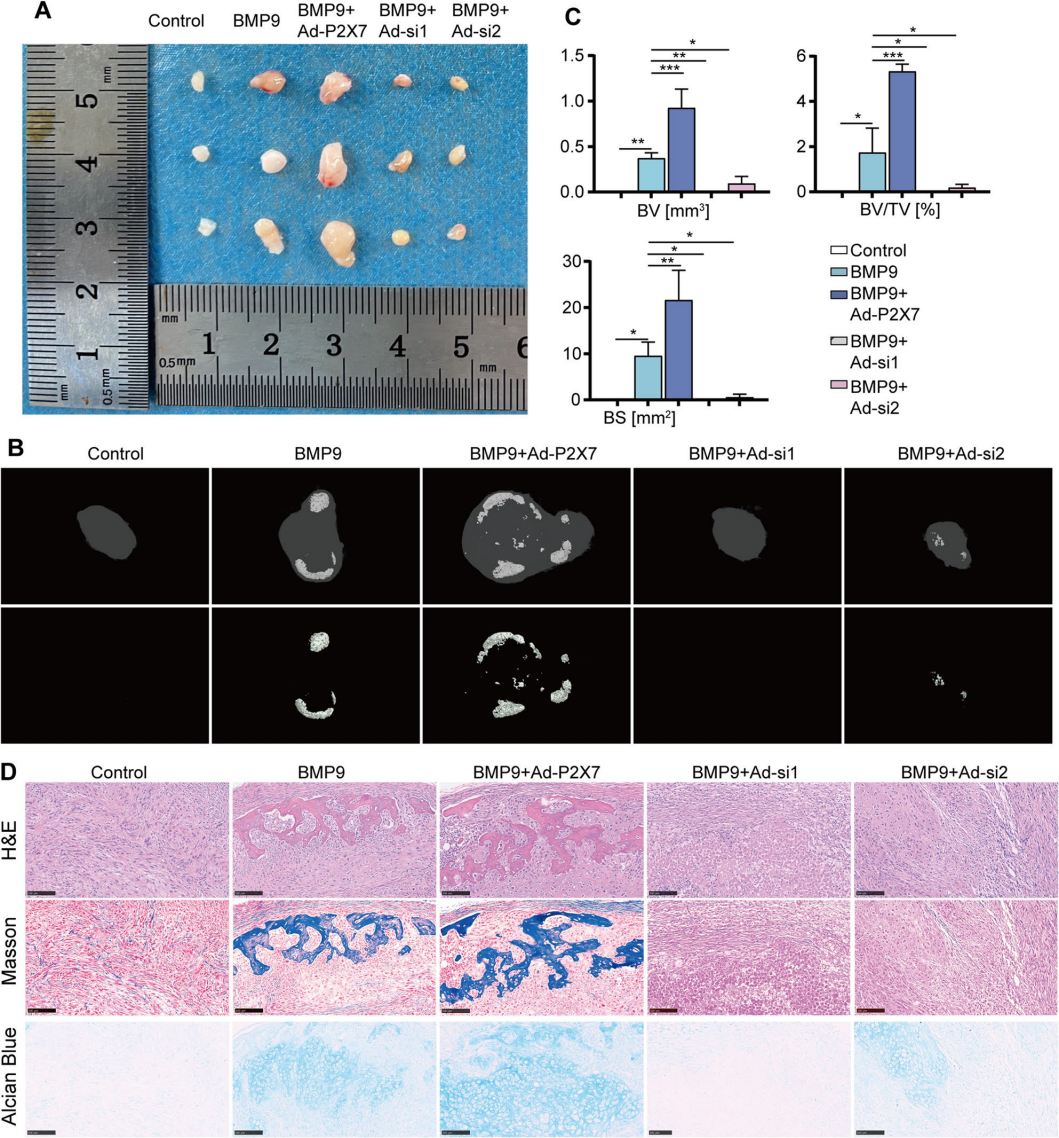
P2X7 promotes BMP9-induced MSCs ectopic bone formation in nude mice.** A** The grass images of subcutaneous masses formed in each treatment group. **B** Micro-CT scan of subcutaneous bone masses in each treatment group. **C** Statistical analysis of BV, BV/TV and BS in each treatment group. **D** H&E staining, Masson staining and Alcian blue staining to assess the amount and maturity of the bone formed in each treatment group. Scale bars, 100 μm. *P < 0.05; **P < 0.01; ***P < 0.001

We then further verify the regulatory role of P2X7 in BMP9’s osteo-induction function in vivo by adopting the classical ectopic (subcutaneous) osteogenesis model in nude mice. Micro-CT analysis demonstrated that P2X7 overexpression potentiated BMP9-induced ectopic bone formation of MSCs, as evidenced by further increase of subcutaneous osteoid mass and bone mass (Fig. 4A and B), as well as higher levels of bone volume (BV), bone volume/total volume (BV/TV) and bone surface (BS) (Fig. 4C). In contrast, si-P2X7 obviously suppresses BMP9-induced ectopic bone formation of MSCs (Fig. 4A-C). Histological staining revealed that compared with the BMP9 alone, the P2X7/BMP9 combination treatment group exhibited an increase in newly-generated trabecular bone and cartilage matrix (Fig. 4D). Conversely, si-P2X7 + BMP9 co-treatment group produced completely opposite effect. These findings indicate that the in vivo osteogenic induction function of BMP9 is also influenced by P2X7, and P2X7 as an essential mediator in the process of BMP9-induced osteogenic differentiation of MSCs.

### Overexpression of P2X7 enhances the repair of bone defects by BMP9, while silencing P2X7 weakens this repair process

**Fig. 5:**
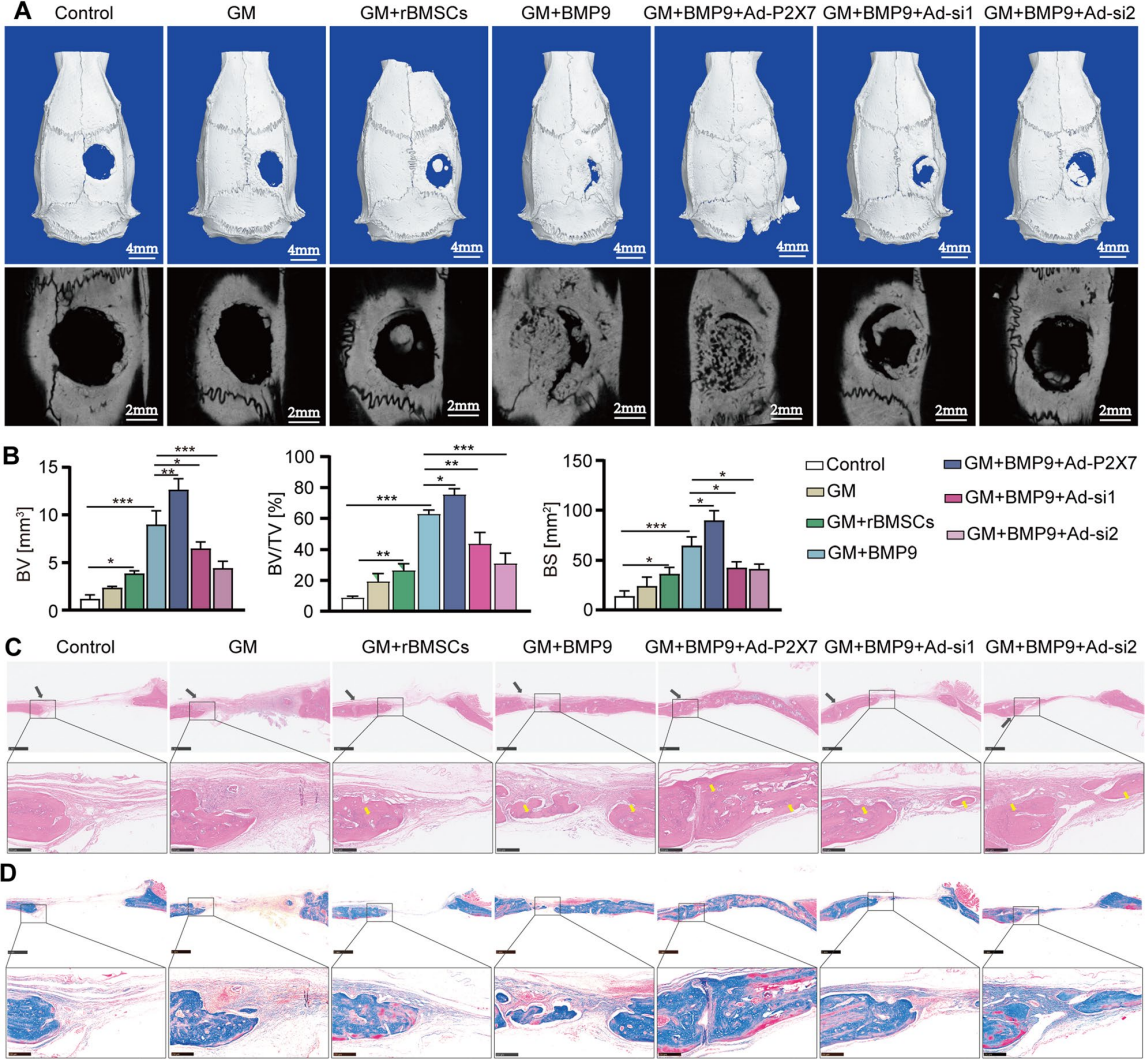
P2X7 promotes the bone repair ability of BMP9 in rat skull defect model.** A** Micro-CT 3D images of skull defect repair in each group. Scale bars, 4 mm. **B** Statistical analysis of BV, BV/TV and BS in different treatment groups. **C** H&E staining of skull samples from each treatment group. Scale bars, 1–250 μm. **D** Mason staining of skull samples from each treatment group. Scale bars, 1–250 μm. *P < 0.05; **P < 0.01; ***P < 0.001

BMP9 has been proven to effectively repair bone defects in animals [26, 27]. Therefore, we decided to ascertain the role of P2X7 in the repair of bone defects by BMP9. rBMSCs were isolated and underwent identification (Fig.S5A), and the safety of the GelMa (GM) scaffold material on these cells was verified (Fig.S5B-E). Subsequently, ALP staining results indicated that P2X7 exerted mediating effects on BMP9-induced osteogenesis in rBMSCs (Fig.S5F), similar to the observations of hBMSCs (Fig.S6A) and other types of MSCs in this study. In the skull defect repair model, P2X7 was observed to further strengthen the repair function of BMP9, as evidenced by a significant improvement in BV, BV/TV and BS on Micro-CT. In addition, there was an increase in bone maturity observed in tissue staining. Conversely, P2X7 silencing inhibited the repairing effect of BMP9 (Fig. 5A-D). Overall, these complementary in vivo models provide conclusive evidence that P2X7 serves as a powerful downstream mediator of BMP9-driven osteogenesis.

### P2X7 regulates BMP9-induced osteogenic differentiation of MSCs through CaMKII

Next, we decided to dissect the molecular mechanism by which P2X7 regulated BMP9-induced osteogenic differentiation of MSCs. Given its established role in Ca²⁺ signaling [28], we measured the calcium level and found that BMP9 promoted the intracellular calcium level in MSCs (Fig.S7A). This effect was significantly enhanced by either overexpressing P2X7 or treating cells with the P2X7 activator BzATP (Fig.S7B). Conversely, silencing of P2X7, P2X7-Δ1/90, or P2X7 inhibitor A740003 attenuated this response (Fig.S8A, B). These results indicate a positive relationship between P2X7 activity and calcium mobilization in MSCs following BMP9 treatment.

**Fig. 6:**
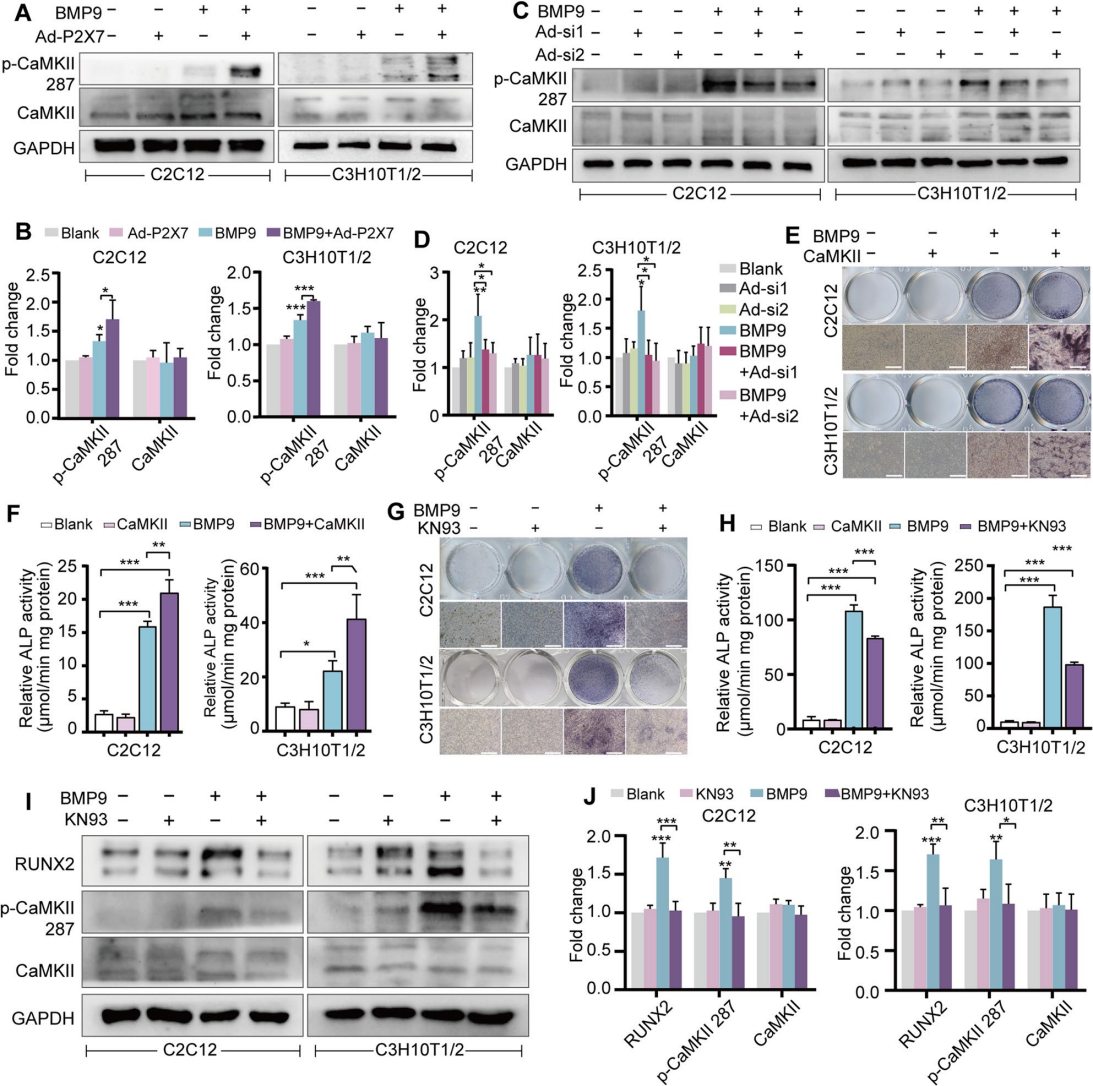
P2X7 mediates BMP9-driven osteogenic differentiation of MSCs via CaMKII. **A** Western blot analysis to detect CaMKII phosphorylation in BMP9-treated MSCs after P2X7 overexpression. **B** Quantitative analysis of Western blot results in Fig. 6A. **C** Western blot to detect CaMKII phosphorylation in BMP9-treated MSCs after P2X7 silencing. **D** Quantitative analysis of Western blot results in Fig. 6C. **E** ALP staining to assess the osteogenic capacity of BMP9-treated MSCs after CaMKII overexpression. Scale bars, 100 μm. **F** Quantification of ALP activity to assess the osteogenic capacity of BMP9-treated MSCs after CaMKII overexpression. **G** ALP staining to assess the osteogenic capacity of BMP9-treated MSCs in the presence of CaMKII inhibitor KN93. Scale bars, 100 μm. **H** ALP activity quantification to assess the osteogenic capacity of BMP9-treated MSCs in the presence of CaMKII inhibitor KN93 (**I**) Western blot analysis to detect RUNX2 protein level and CaMKII phosphorylation in MSCs after BMP9 treatment in the presence of KN93. **J** Quantitative analysis of the Western blot results in Fig. 6I. *P < 0.05; **P < 0.01; ***P < 0.001

As CaMKII fulfills the role of a primary intracellular calcium sensor [29], we spontaneously thought to determine whether CaMKII is affected by BMP9 and/or P2X7. BMP9 was found to increase the phosphorylation of CaMKII at Threonine 287, with this effect being further enhanced by P2X7 overexpression and suppressed by P2X7 silencing (Fig. 6A-D). Functional studies confirmed the critical role of CaMKII, as CaMKII overexpression amplified BMP9-induced ALP activity, while the specific inhibitor KN93 abolished this enhancement (Fig. 6E-H). Correspondingly, treatment with KN93 reduced both CaMKII phosphorylation and the protein level of the osteogenic master regulator RUNX2 (Fig. 6I, J). The present findings suggest that P2X7 may influence BMP9-induced osteogenic differentiation of MSCs through activating the calcium messenger CaMKII.

### CaMKII interacts with GSK-3β to stabilize β-catenin

**Fig. 7:**
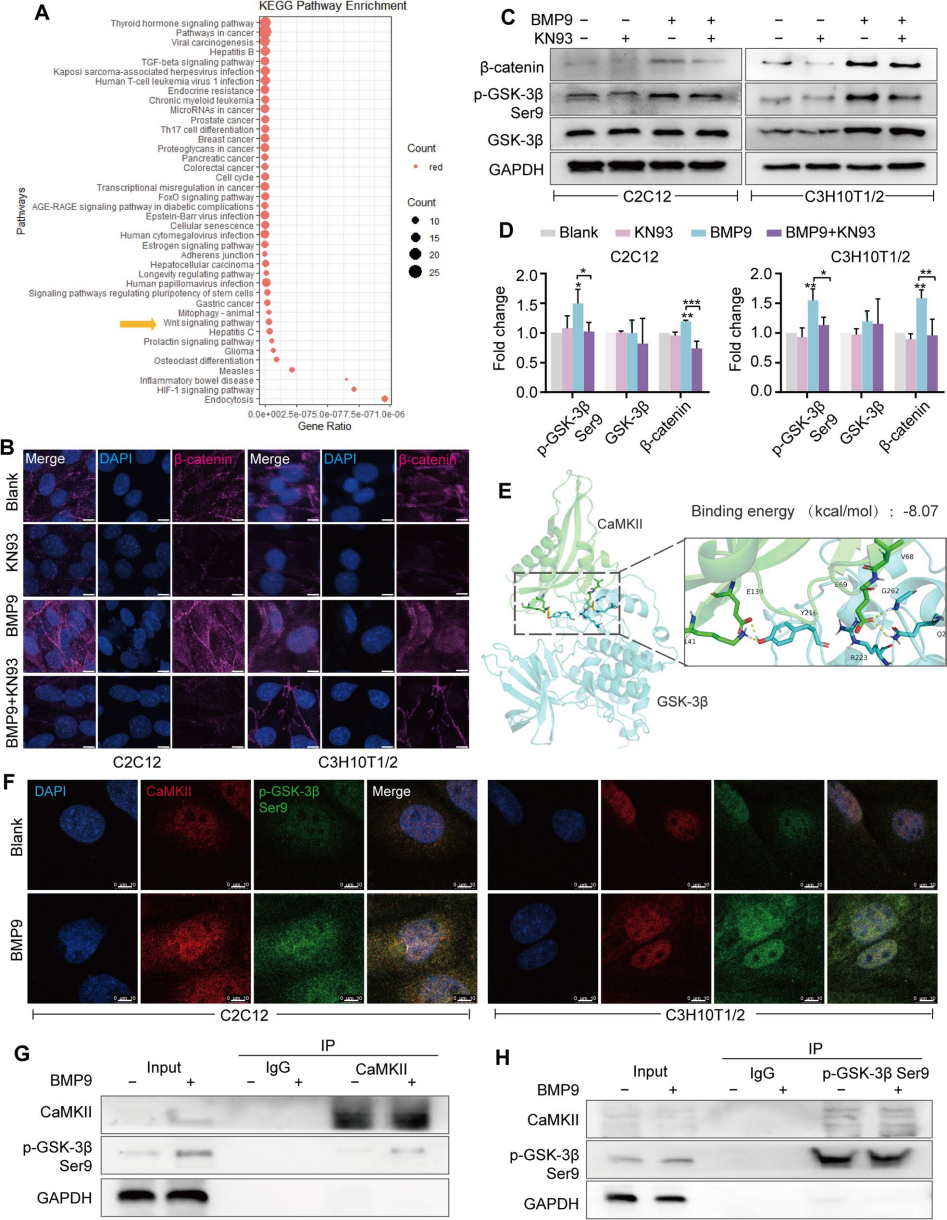
CaMKII stabilizes β-catenin protein by interacting with GSK-3β and promoting the phosphorylation of GSK-3β at Ser9.** A** KEGG analysis to identify the important intersectional signaling pathways between BMP9 and CaMKII in MSCs. **B** Immunofluorescence staining to detect the changes of intracellular β-catenin protein in BMP9-treated MSCs in the presence of KN93. Scale bars, 10 μm. **C** Western blot to detect the alterations in the Wnt/β-catenin signaling pathway in BMP9-treated MSCs in the presence of KN93. **D** Quantitative analysis of the Western blot results in Fig. 7C. **E** Molecular docking simulation of protein-protein interactions between GSK-3β and CaMKII. **F** Immunofluorescence staining to show the colocalization of CaMKII and p-GSK-3β Ser9 in BMP9-treated MSCs. Scale bars, 10 μm. **G** The interaction between CaMKII and GSK-3β was analyzed by IP with CaMKII antibody and Western blot. **H** The interaction between CaMKII and GSK-3β was analyzed by IP with p-GSK-3β Ser9 antibody and Western blot. *P < 0.05; **P < 0.01; ***P < 0.001

To elucidate the mechanistic link between CaMKII activation and BMP9-induced osteogenic differentiation, we utilized STRING to analyze the intersection genes potentially involved in the osteogenic function of BMP9 and CaMKII, and performed KEGG enrichment analysis. Of particular interest was the Wnt/β-catenin signaling pathway because it is a well-established dominant pathway for bone formation (Fig. 7A). Following the inhibition of CaMKII by KN93, BMP9-stimulated MSCs showed impaired intranuclear localization of β-catenin (Fig. 7B), as well as a decrease in total β-catenin protein (Fig. 7C, D). It is worth noting that KN93 also induced a reduction in the phosphorylation of GSK-3β protein at serine 9 residue (Fig. 7C, D). Being an intracellular serine/threonine kinase, GSK-3β negatively determined Wnt/β-catenin signaling activity by promoting β-catenin to be targeted for proteasomal degradation, while the phosphorylation of serine 9 residue may lead to the decrease of GSK-3β kinase activity [30, 31]. Considering its function, we speculated that GSK-3β might act as an intermediary link between β-catenin and CaMKII. Protein-protein docking simulations predicted a strong binding affinity between CaMKII and GSK-3β (Fig. 7E). Next, the prediction was further experimentally confirmed by immunofluorescence staining and immunoprecipitation assay. The results showed that CaMKII co-located and physically interacted with p-GSK-3β, and this colocalization and physical interaction were strengthened under BMP9 treatment (Fig. 7F-H). The present data demonstrate that CaMKII may be involved in maintaining the stability of β-catenin, possibly by interacting with GSK-3β and inducing inhibitory phosphorylation of GSK-3β at Ser 9.

### BMP9 activates the β-catenin pathway through P2X7

**Fig. 8:**
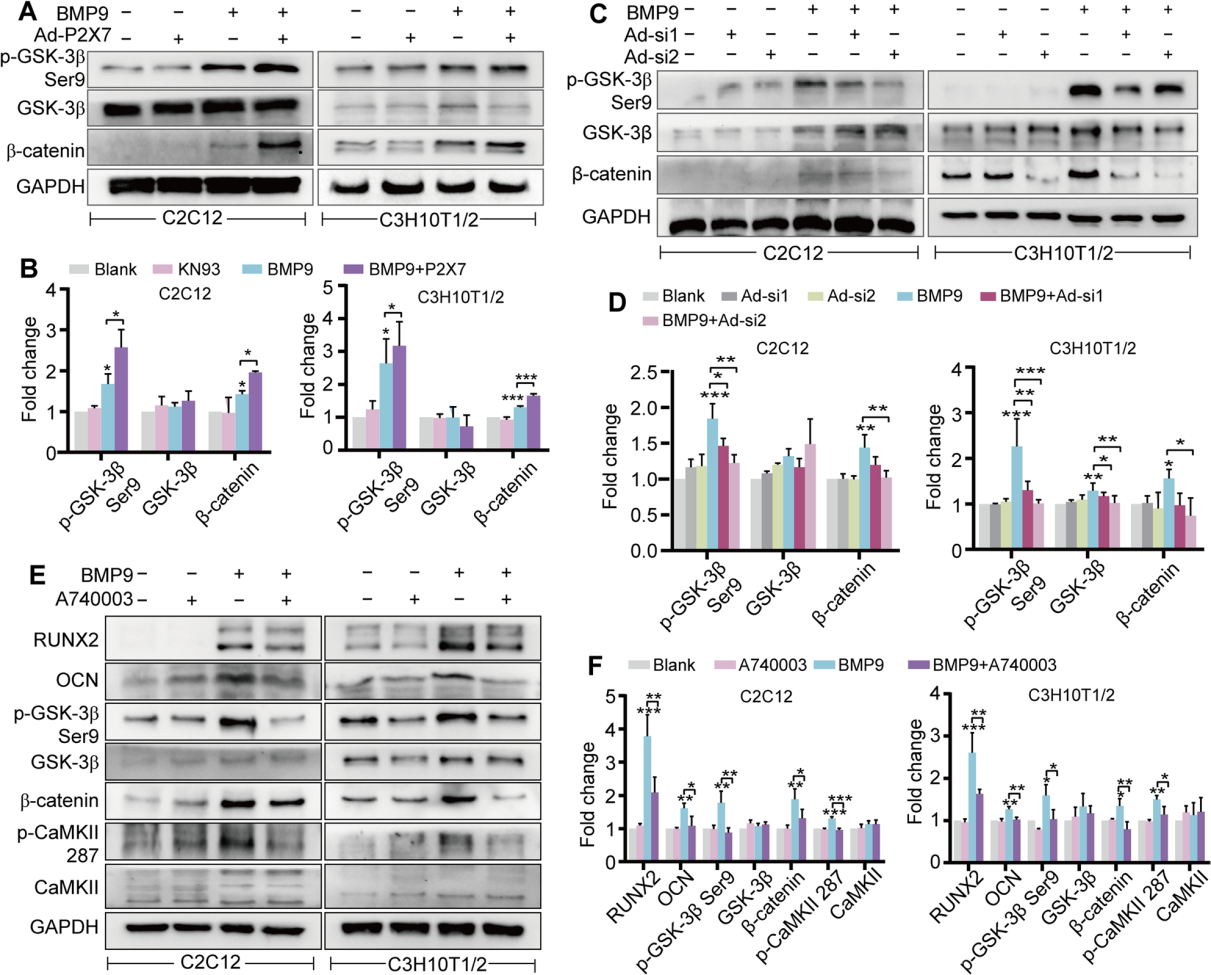
BMP9 activates the β-catenin pathway through P2X7.** A** Western blot to detect the alterations in the Wnt/β-catenin signaling pathway after BMP9 and/or P2X7 overexpression treatment. **B** Quantitative analysis of Western blot results in Fig. 8A. **C** Western blot to analyze the alterations in the Wnt/β-catenin signaling pathway after BMP9 and/or P2X7 silencing treatment. **D** Quantitative analysis of Western blot results in Fig. 8C. **E** Western blot to detect the alterations in osteogenic markers, the Wnt/β-catenin signaling pathway and CaMKII phosphorylation after BMP9 treatment in the presence of A740003. **F** Quantitative analysis of Western blot results in Fig. 8E. *P < 0.05; **P < 0.01; ***P < 0.001

As demonstrated by the aforementioned results, BMP9 activated the Wnt/β-catenin signaling pathway by increasing the interaction between CaMKII and GSK-3β, leading to inhibitory phosphorylation of GSK-3β and subsequent β-catenin stabilization. We then examined whether P2X7 participated in BMP9-activated Wnt/β-catenin signaling. As anticipated, P2X7 overexpression in BMP9-treated MSCs significantly prolonged β-catenin protein stability and enhanced GSK-3β phosphorylation at Ser9 compared to BMP9 treatment alone (Fig. 8A and B). Conversely, silencing of P2X7 markedly reduced β-catenin accumulation (Fig. 8C and D). Inhibition of P2X7 with A740003 not only attenuated the promoting effect of BMP9 on osteogenic markers expression, but also diminished CaMKII phosphorylation and subsequent downstream events including GSK-3β (Ser9) phosphorylation and β-catenin stabilization (Fig. 8E and F). Overall, these results support a novel mechanism whereby BMP9 up-regulates P2X7 expression and potentiates P2X7-mediated calcium influx to activate CaMKII, which then phosphorylates GSK-3β at Ser9 to inhibit β-catenin degradation, ultimately driving osteogenic differentiation of MSCs through enhanced β-catenin signaling.

## Discussion

Even though BMP9 is not as well-researched as other BMPs family members, it has been demonstrated to possess robust potential in guiding MSCs to differentiate into osteoblast. In the present study, we demonstrated that the P2X7 purinergic receptor may function as an essential mediator in BMP9-driven osteogenic differentiation of MSCs. The most critical physiological function of P2X7 is to mediate calcium ion influx. In the skeletal system, calcium is the primary component of the mineralized bone matrix, endowing bones with their hardness and strength. Calcium contributes greatly to the structural integrity of bone, and directly facilitates the osteogenic differentiation of MSCs through multiple signaling pathways [32]. In addition, calcium ions can potentiate the responsiveness of MSCs to BMP/Smad signaling by stimulating the phosphorylation and nuclear translocation of Smad1/5/8, thereby further boosting the differentiation of MSCs into the osteoblast lineage [33]. Calcium supplementation can enhance the osteogenic potential of MSCs, but impede adipogenesis in these cells at the same time [34]. Therefore, calcium preparation has been employed as a pharmaceutical agent in clinical setting to promote bone formation. The present study revealed that BMP9 significantly up-regulated P2X7 expression via Smad signaling, and changes in P2X7 expression can also affect BMP9 induced osteogenic differentiation and calcium flow. Meanwhile, our results indicate that P2X7 induction is not limited to BMP9, BMP2 and BMP4 can also stimulate its changes. This may be related to the co-activation of SMAD signaling pathways by BMP family members.

Calcium-triggered signaling is intricate and diverse, involving the interaction and cascade of various proteins, channels and signal molecules [35]. Among these, calcium (Ca^2+^)/calmodulin (CaM)-dependent kinase II (CaMKII) acts as a central hub by directly sensing intracellular Ca²⁺ fluctuations and orchestrating diverse physiological cellular responses [29]. When calcium and CaM bind together to form a complex, CaMKII undergoes autophosphorylation. The self-phosphorylation of CaMKII at Threonine 286 (T286) or T305/306 directly affects CaMKII’s kinase activity by causing local changes in its conformation [35]. However, CaMKII phosphorylation at T287 leads to another interesting effect, known as CaM trapping. CaM trapping can increase the binding affinity of CaM by up to 1000-fold, which aids in maintaining the Ca^2+^/CaM complex intact and sustaining CaMKII activity even when Ca^2+^ levels are low [36]. The current study proved that BMP9 could activate CaMKII through P2X7. Not only that, activation or inhibition of CaMKII yielded a corresponding promotion or suppression of BMP9-induced osteogenic differentiation in MSCs. Certain biomolecules including N-formylmethionyl-leucyl-phenylalanine (fMLP) and dentin phospholipids, or physical stimuli such as electric fields have been reported to activate CaMKII, thus promoting the osteogenic differentiation of MSCs [37, 38]. Nonetheless, the precise pattern and detailed molecular mechanisms of CaMKII in the differentiation of MSCs into the osteoblast lineage are still far from being thoroughly comprehended. Consequently, it is imperative to further define the key downstream signals and molecules that crucially participate in CaMKII-mediated BMP9-induced MSCs osteogenic differentiation.

By performing KEGG analysis, we identified the important intersectional signaling pathways between BMP9 and CaMKII, which encompassed the Wnt/β-catenin pathway. The Wnt/β-catenin pathway occupies a central position in the development of tissues and organs, influencing cell proliferation and differentiation, and determining tissue structure and growth [39, 40]. In the skeletal system, it serves as a pivotal signal in controlling bone development and formation [32]. Importantly, the Wnt/β-catenin signaling has been clearly validated to be indispensable for BMP9-induced osteogenic differentiation of MSCs derived from different sources [13]. During the process of BMP9-induced osteogenic differentiation of MSCs, β-catenin emerges as a potent inducer, with its activity being regulated by both PDK4 and Smad1/5/8 signaling [41, 42]. Here, we identified β-catenin as a novel downstream effector of CaMKII, and the phosphorylation of GSK-3β at serine 9 is the crucial step that link them together. GSK-3β is an essential component of the β-catenin disruption complex, where it fulfills a regulatory function by promoting the phosphorylation of β-catenin, thereby facilitating its degradation. Following phosphorylation by GSK-3β, β-catenin is readily identifiable and ubiquitinated by β-Trcp (an E3 ubiquitin ligase) for degradation [43]. The phosphorylation of GSK3β itself seriously affects its kinase activity. Nonetheless, it is important to recognize that phosphorylation at different sites may produce opposite effects [44]. Crucially, the phosphorylation of GSK-3β at Ser9 may potently inhibit its kinase activity, thus weakening its promoting effect on the degradation of β-catenin [45, 46]. We revealed that BMP9 increased both the protein level of β-catenin and the phosphorylation of GSK-3β at Ser9, suggesting the activation of Wnt/β-catenin signaling. Additionally, previous studies have shown that P2X7 can enhance Wnt/β-catenin protein signaling in osteoblasts [47]. In this study, we further confirmed that overexpression or knockdown of P2X7 leads to enhanced or reduced activation of Wnt/β-catenin signaling triggered by BMP9. Furthermore, after administration of the CaMKII inhibitor KN93, the phosphorylation level of CaMKII inhibited by BMP9 in MSCs decreased, and the phosphorylation level of GSK-3β at Ser9 also decreased, suggesting a possible association between CaMKII and GSK-3β. The interaction between CaMKII and GSK-3β has been reported in certain cell models. For instance, CaMKII can associate with and phosphorylate GSK-3β, which is crucial for the survival of depolarization-dependent neurons [48]. The HIV-1 protein Tat has been observed to induce cell death in immature oligodendrocytes by facilitating CaMKII/GSK-3β interaction [49]. In this study, protein - protein docking was employed to predict the interaction between CaMKII and GSK‑3β, and co‑immunoprecipitation (Co‑IP) assays further confirmed their binding. However, whether CaMKII and GSK‑3β interact directly or through intermediate proteins remains to be elucidated by additional experiments.

## Conclusions

In summary, this study demonstrates that the involvement of P2X7 is essential in the osteogenic differentiation of MSCs induced by BMP9, potentially through the CaMKII/GSK-3β/β-catenin signaling axis. This ultimately establishes P2X7 as a critical molecular link between BMP9/Smad signaling and calcium-related osteogenic commitment. The discovery not only reveals a new mechanism by which BMP9 drives osteogenic differentiation of MSCs, but may also provide new insights for the future clinical application of BMP9 in the treatment of bone defects.

## Supplementary Information

Supplementary Material 1.

Supplementary Material 2.

Supplementary Material 3.

Supplementary Material 4.

Supplementary Material 5.

Supplementary Material 6.

Supplementary Material 7.

Supplementary Material 8.

Supplementary Material 9.

Supplementary Material 10.

Supplementary Material 11.

Supplementary Material 12.

## Data Availability

No datasets were generated or analysed during the current study.
