# Supplementary figures and images for: P2X7 is an important mediator of BMP9-induced osteogenic differentiation of mesenchymal stem cells
Source: Cell Commun Signal. 2026 Feb 25;24:204. doi: 10.1186/s12964-026-02747-w (PMC13041228; doi:10.1186/s12964-026-02747-w)

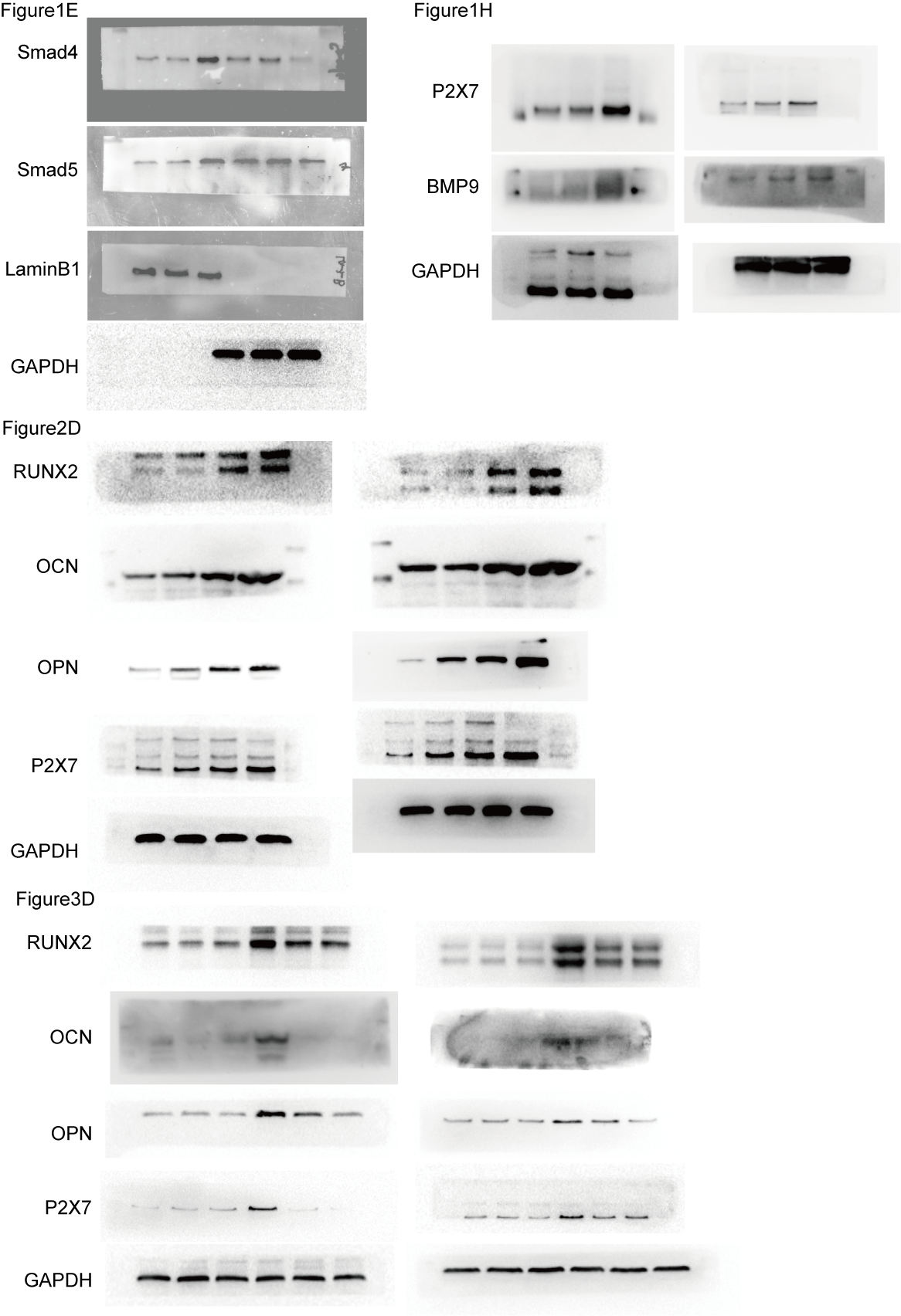


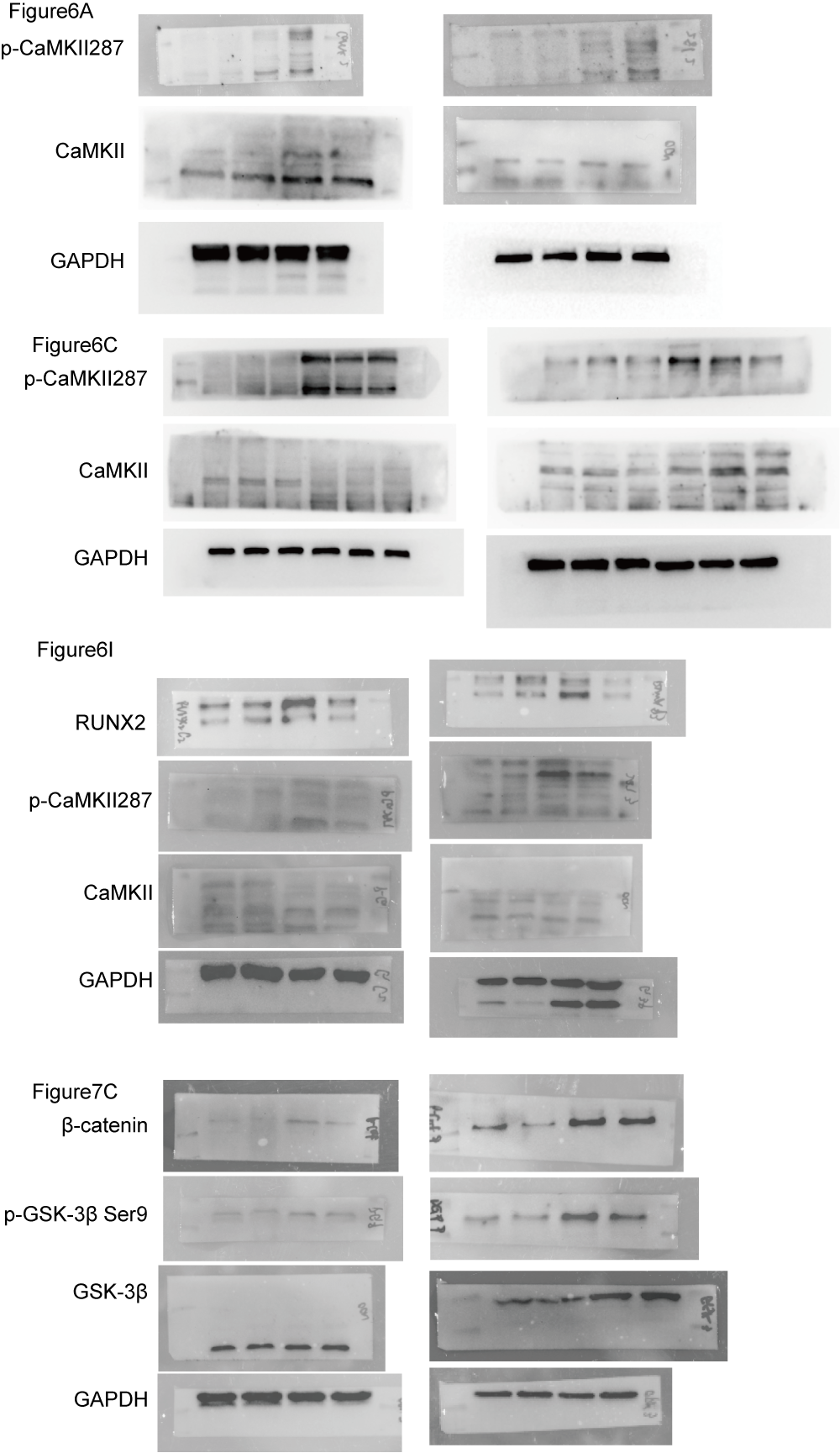


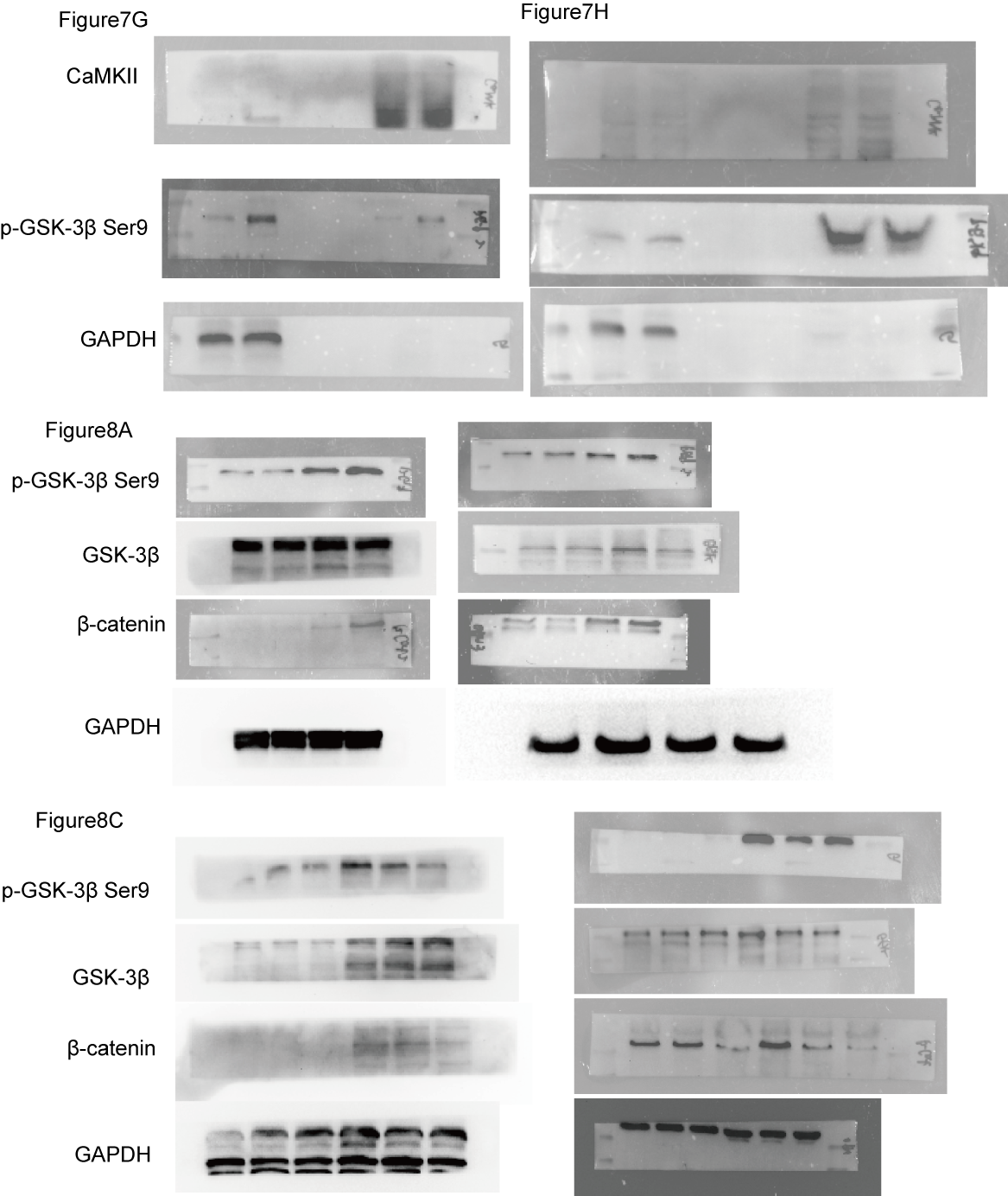


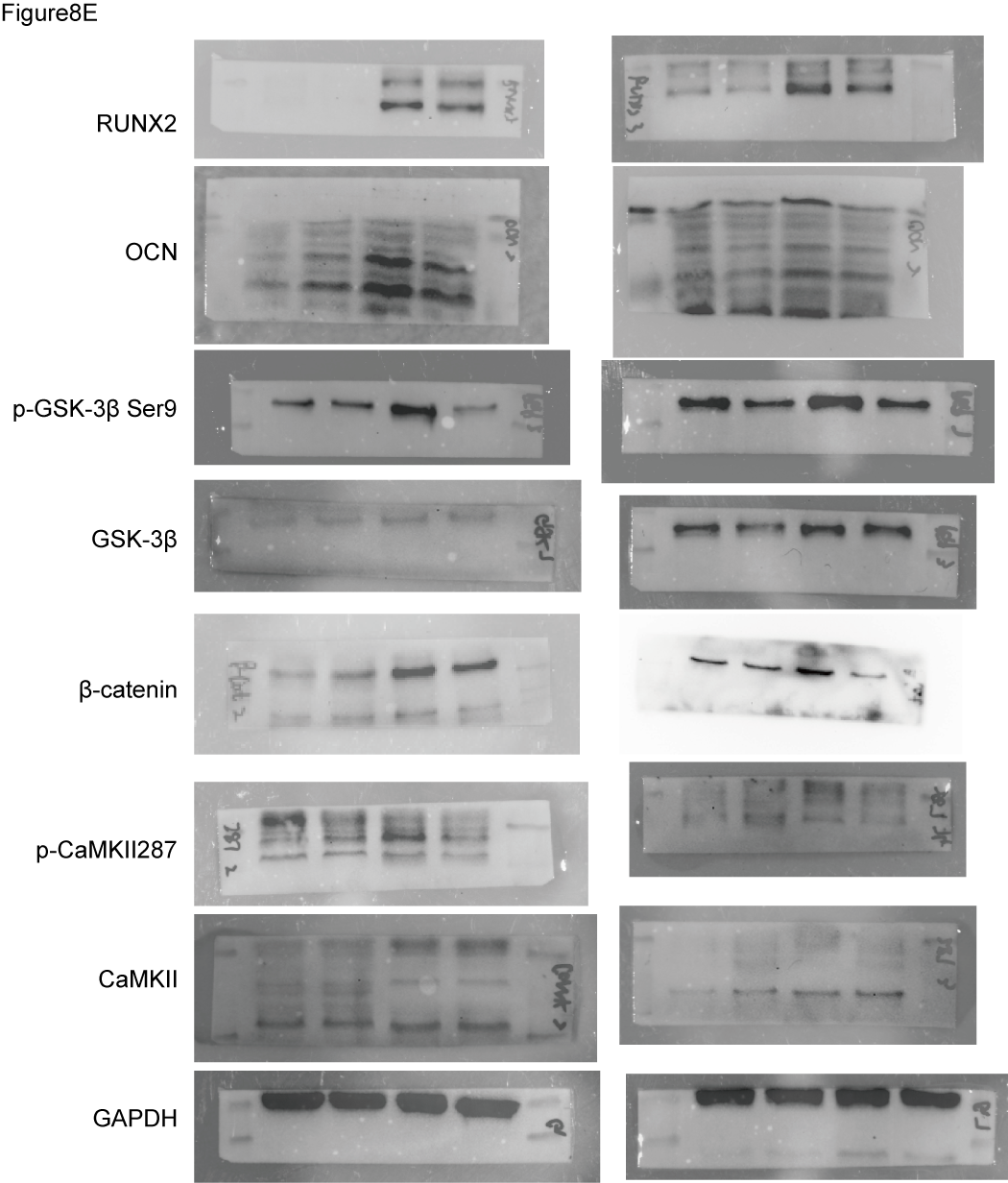

Supplement: Supplementary file 2 — Supplementary Material 2. [file 12964_2026_2747_MOESM2_ESM.docx]

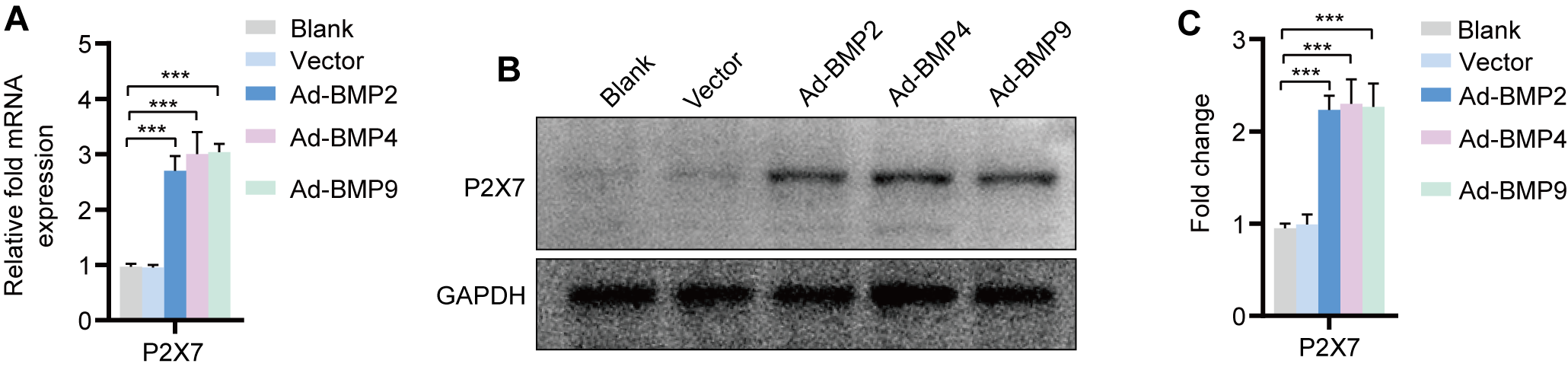

Supplement: Supplementary file 4 — Supplementary Material 4. [file 12964_2026_2747_MOESM4_ESM.tif]

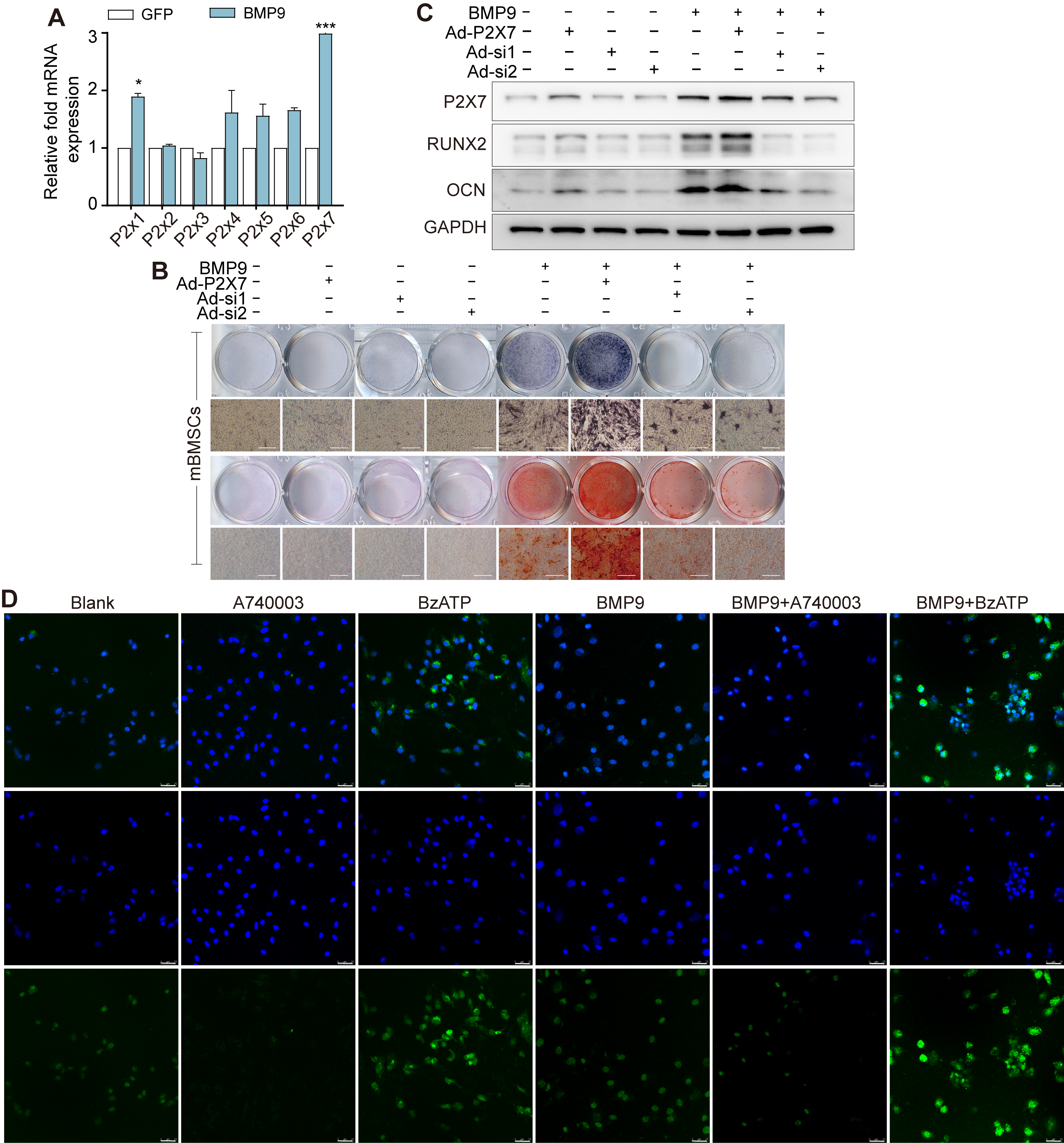

Supplement: Supplementary file 5 — Supplementary Material 5. [file 12964_2026_2747_MOESM5_ESM.tif]

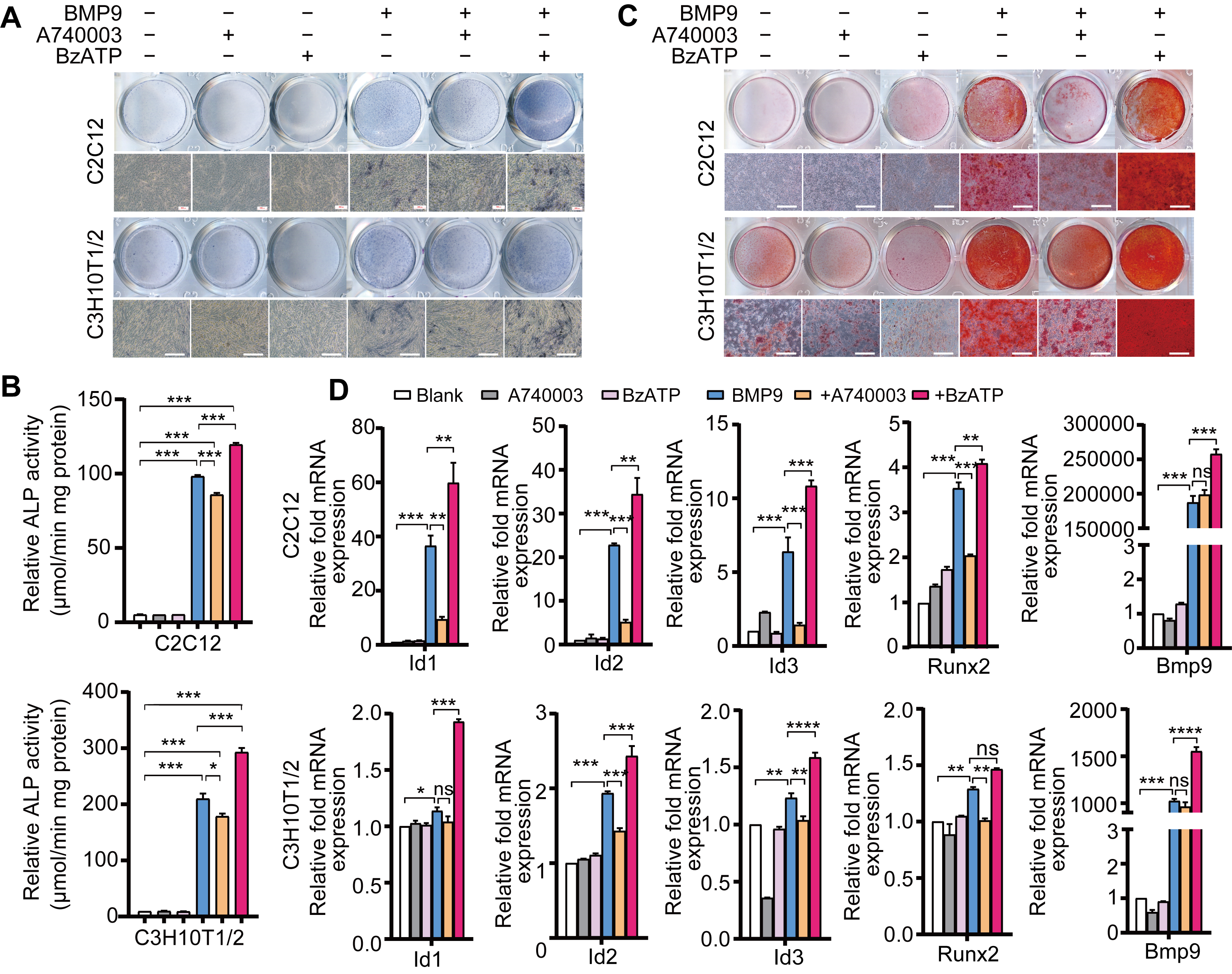

Supplement: Supplementary file 6 — Supplementary Material 6. [file 12964_2026_2747_MOESM6_ESM.tif]

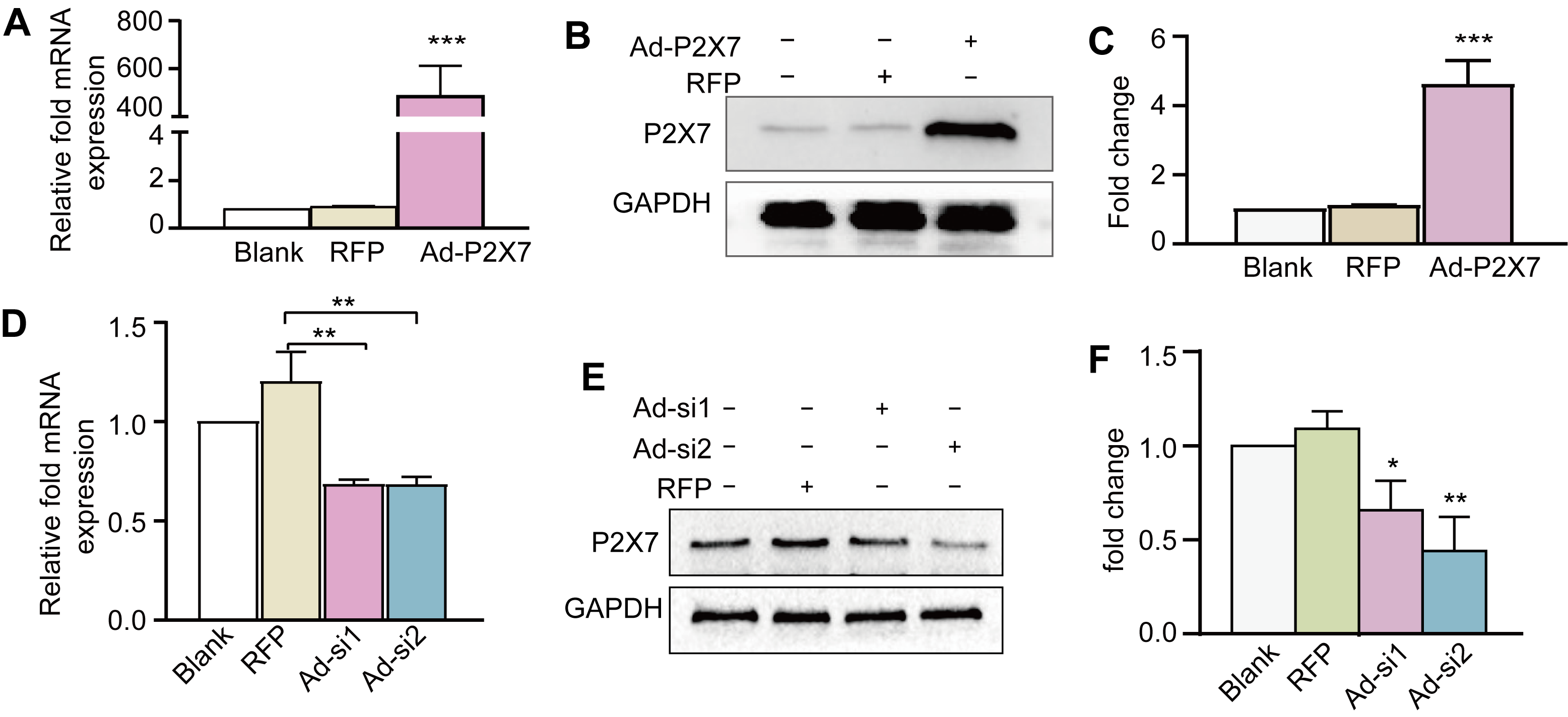

Supplement: Supplementary file 7 — Supplementary Material 7. [file 12964_2026_2747_MOESM7_ESM.tif]

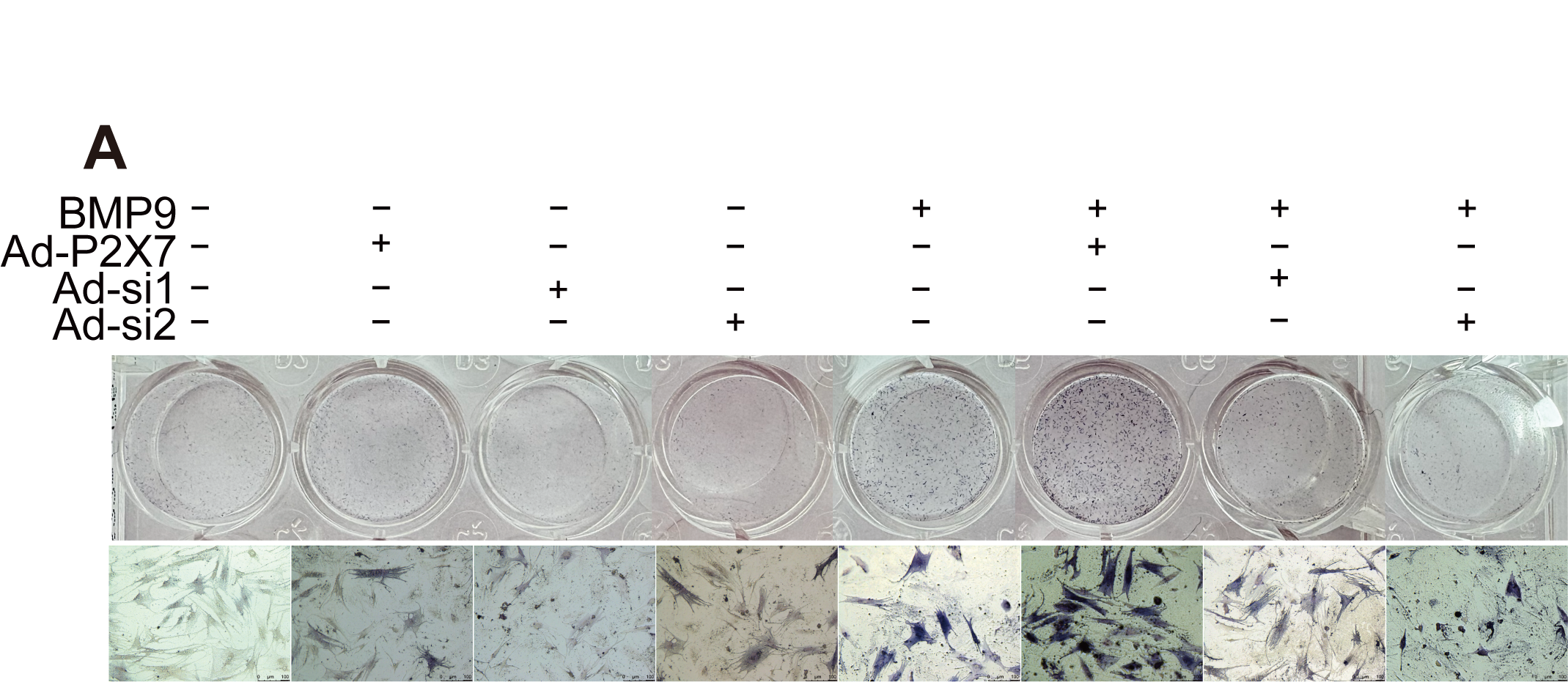

Supplement: Supplementary file 8 — Supplementary Material 8. [file 12964_2026_2747_MOESM8_ESM.tif]

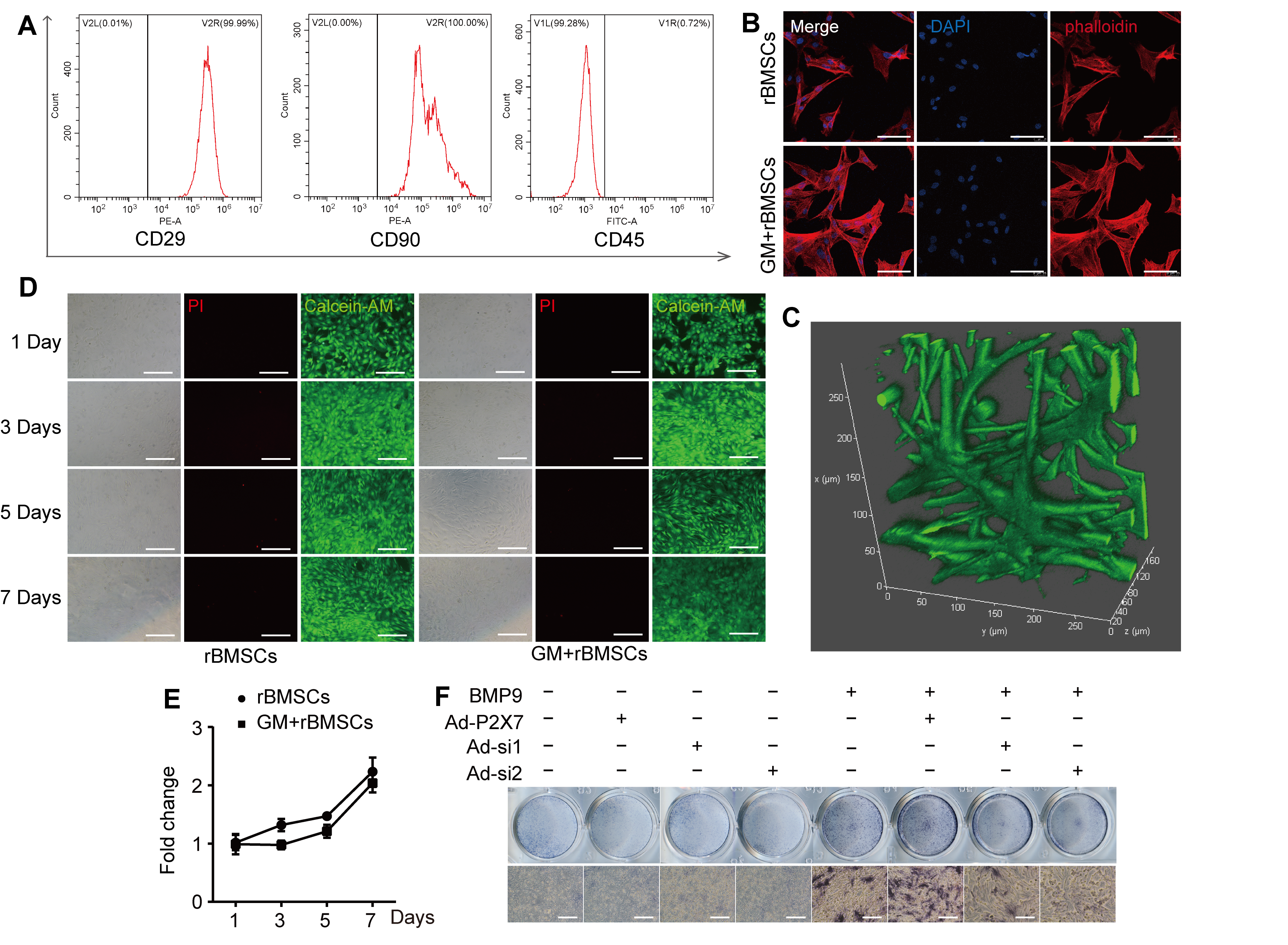

Supplement: Supplementary file 9 — Supplementary Material 9. [file 12964_2026_2747_MOESM9_ESM.tif]

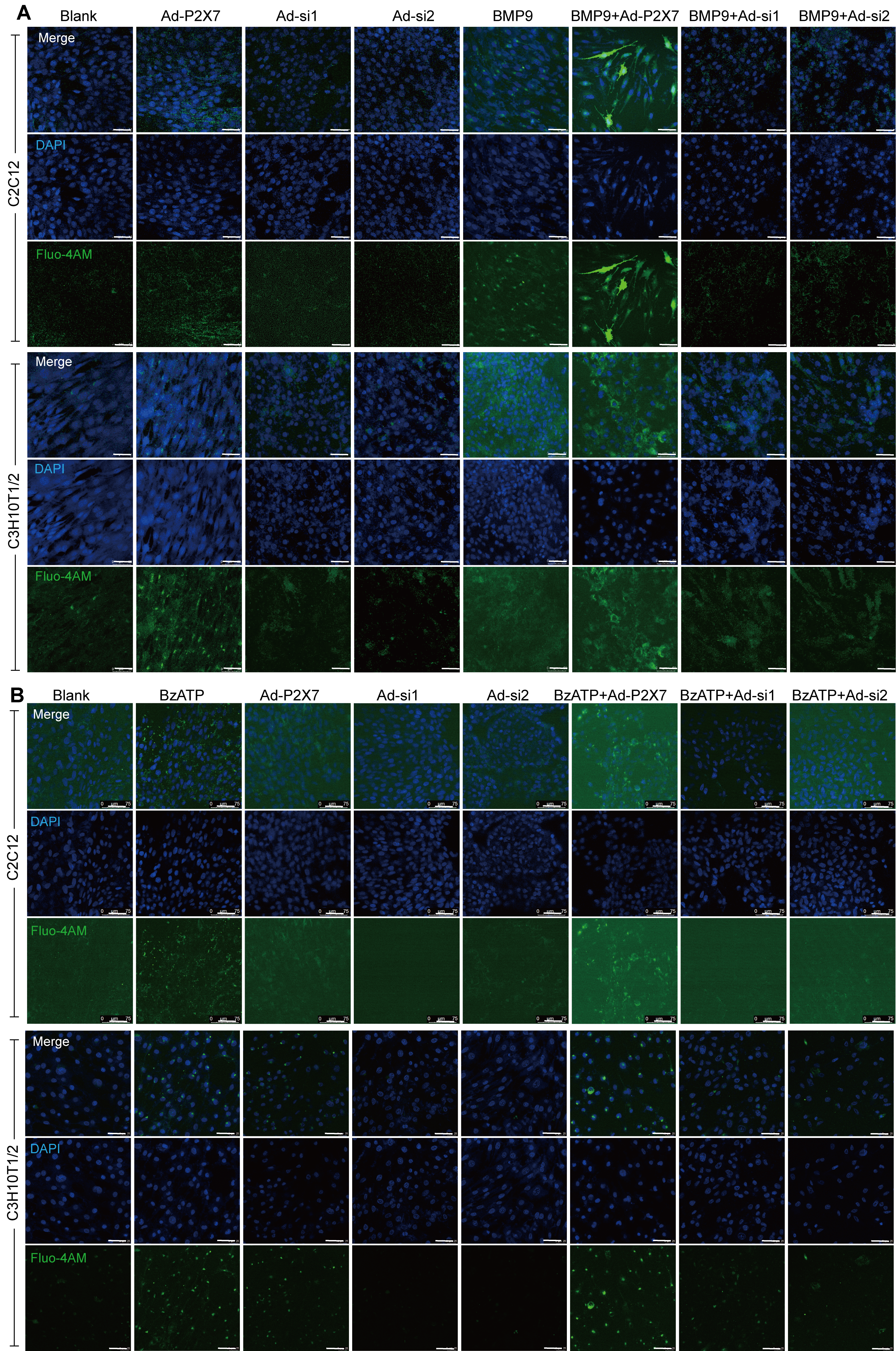

Supplement: Supplementary file 10 — Supplementary Material 10. [file 12964_2026_2747_MOESM10_ESM.tif]

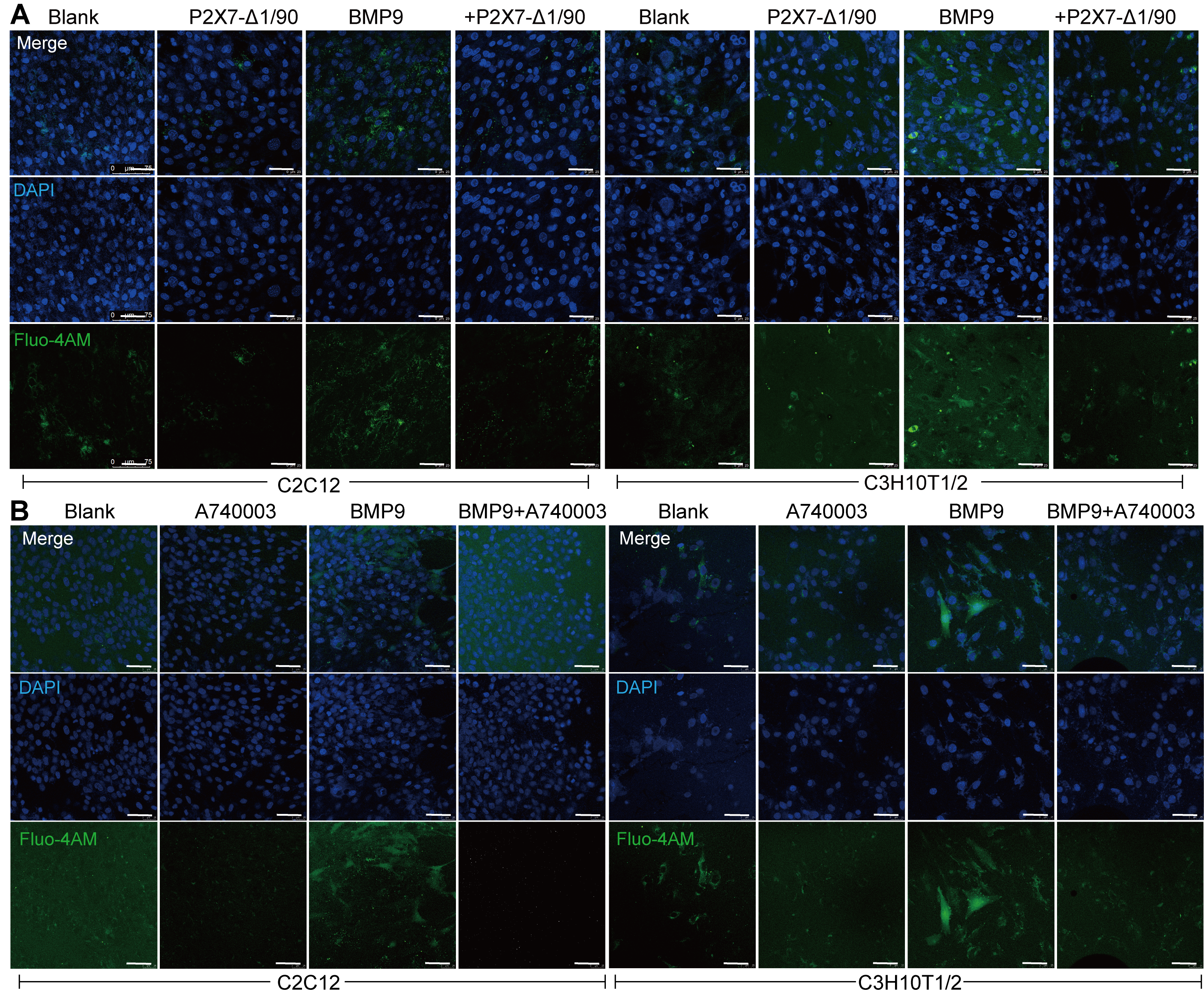

Supplement: Supplementary file 11 — Supplementary Material 11. [file 12964_2026_2747_MOESM11_ESM.tif]
