# Supplementary material for: P2X7 is an important mediator of BMP9-induced osteogenic differentiation of mesenchymal stem cells
Source: Cell Commun Signal. 2026 Feb 25;24:204. doi: 10.1186/s12964-026-02747-w (PMC13041228; doi:10.1186/s12964-026-02747-w)
Supplement: Supplementary file 12 — Supplementary Material 12. [file 12964_2026_2747_MOESM12_ESM.docx]

**
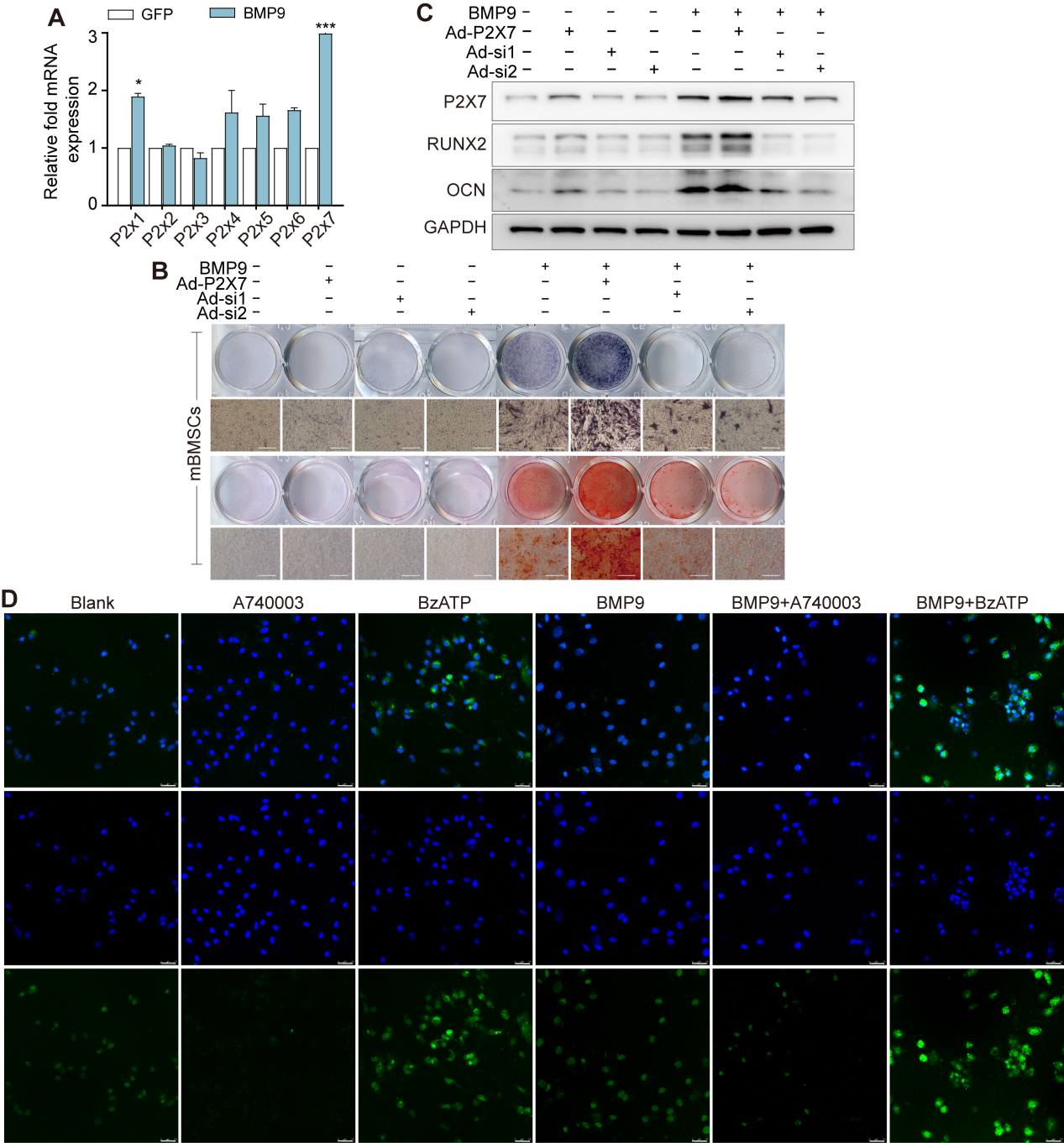
**

**Supplementary Fig. 1 P2X7 promotes the osteogenic induction capacity of BMP9 in primary mBMSCs.** **(A)** qRT-PCR to detect the mRNA levels of P2X members in mBMSCs after BMP9 treatment. **(B)** ALP staining and alizarin red S staining to assess the effect of P2X7 on the osteogenic differentiation of mBMSCs upon BMP9 treatment. Scale bars, 100 μm. **(C)** Western blot assay to evaluate the effect of P2X7 on the protein level of osteogenic molecules in mBMSCs. **(D)** Fluo-4AM probe to analyze the effect of P2X7 on the intracellular Ca^2+^ levels in mBMSCs upon BMP9 stimulation. Scale bars, 25 μm. *P < 0.05, **P < 0.01, ***P < 0.001.


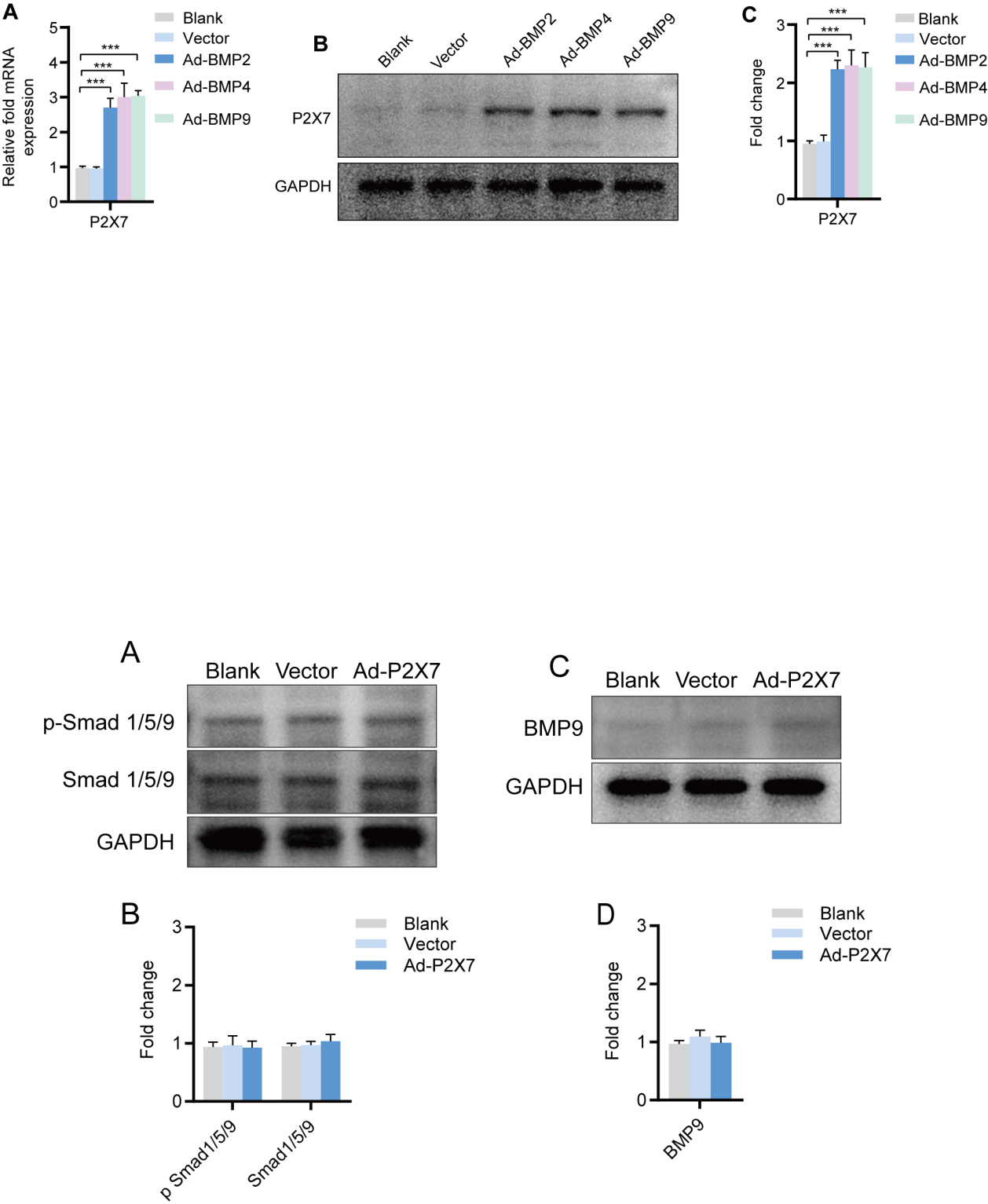


**Supplementary Fig. 2 BMP2 and BMP4 can promote P2X7 transcription and translation.** **(A)** qRT-PCR to evaluate the mRNA levels of P2X7 in C3H10T1/2 after BMP9 treatment. **(B)** Western blot assay to detect the protein level of osteogenic P2X7 in C3H10T1/2. **(C)** Quantitative analysis of Western blot results in **B**. *P < 0.05; **P < 0.01; ***P < 0.001.


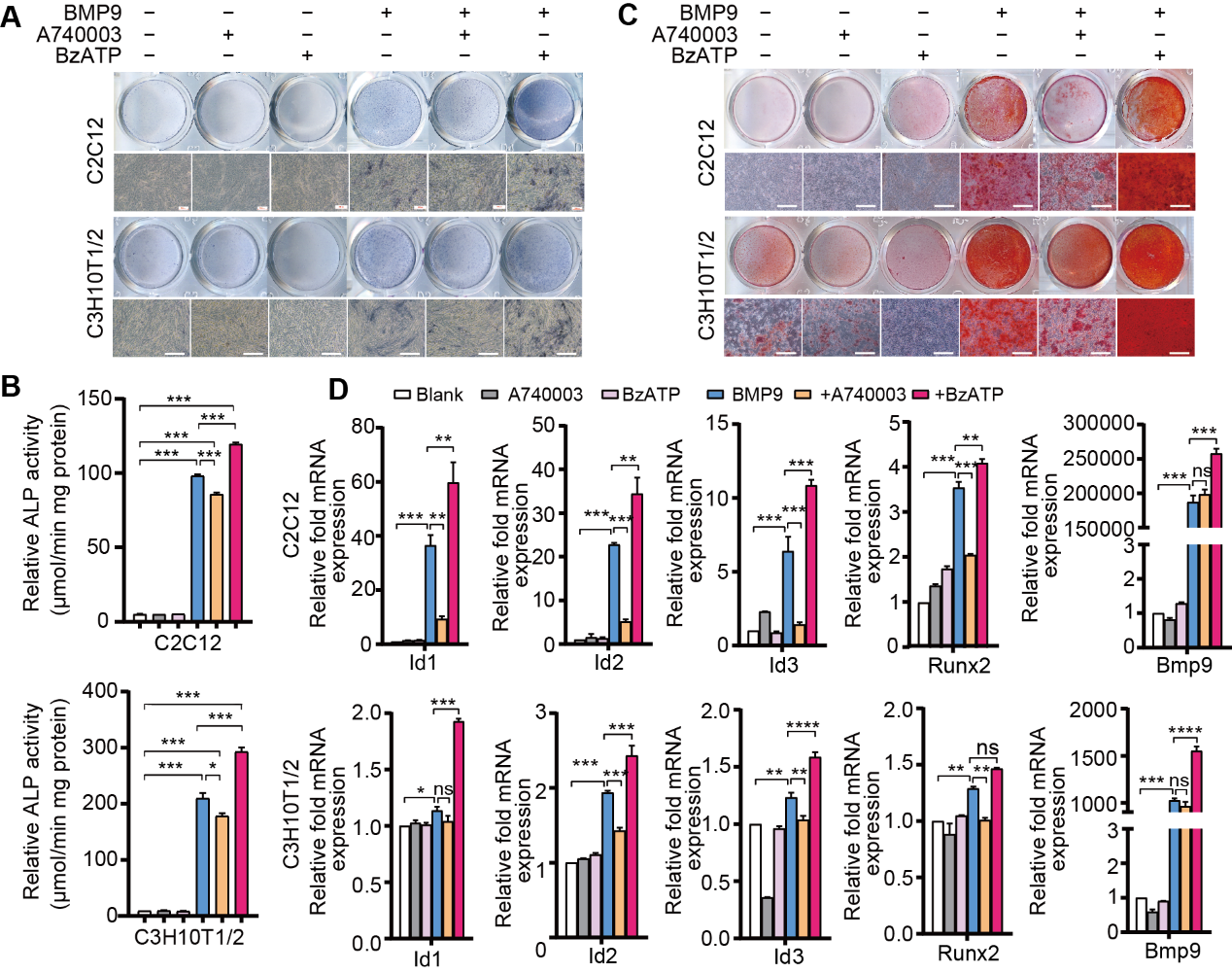


**Supplementary Fig.3 P2X7 regulates BMP9-induced osteogenic differentiation of MSCs. (A)** ALP staining to assess the effect of P2X7 activator BzATP and P2X7 inhibitor A740003 on the early osteogenic differentiation of MSCs upon BMP9 stimulation. Scale bars, 100 μm. **(B)** ALP activity quantification to analyze the effect of BzATP and P A740003 on the early osteogenic differentiation of MSCs upon BMP9 treatment. **(C)** Alizarin red S staining to examine the effect of BzATP and P A740003 on the late osteogenic differentiation of MSCs upon BMP9 treatment. Scale bars, 100 μm. **(D)** qRT-PCR to detect the mRNA levels of osteogenesis-related molecules in MSCs upon BMP9 treatment in the presence of BzATP and P A740003. *P < 0.05, **P < 0.01, ***P < 0.001.


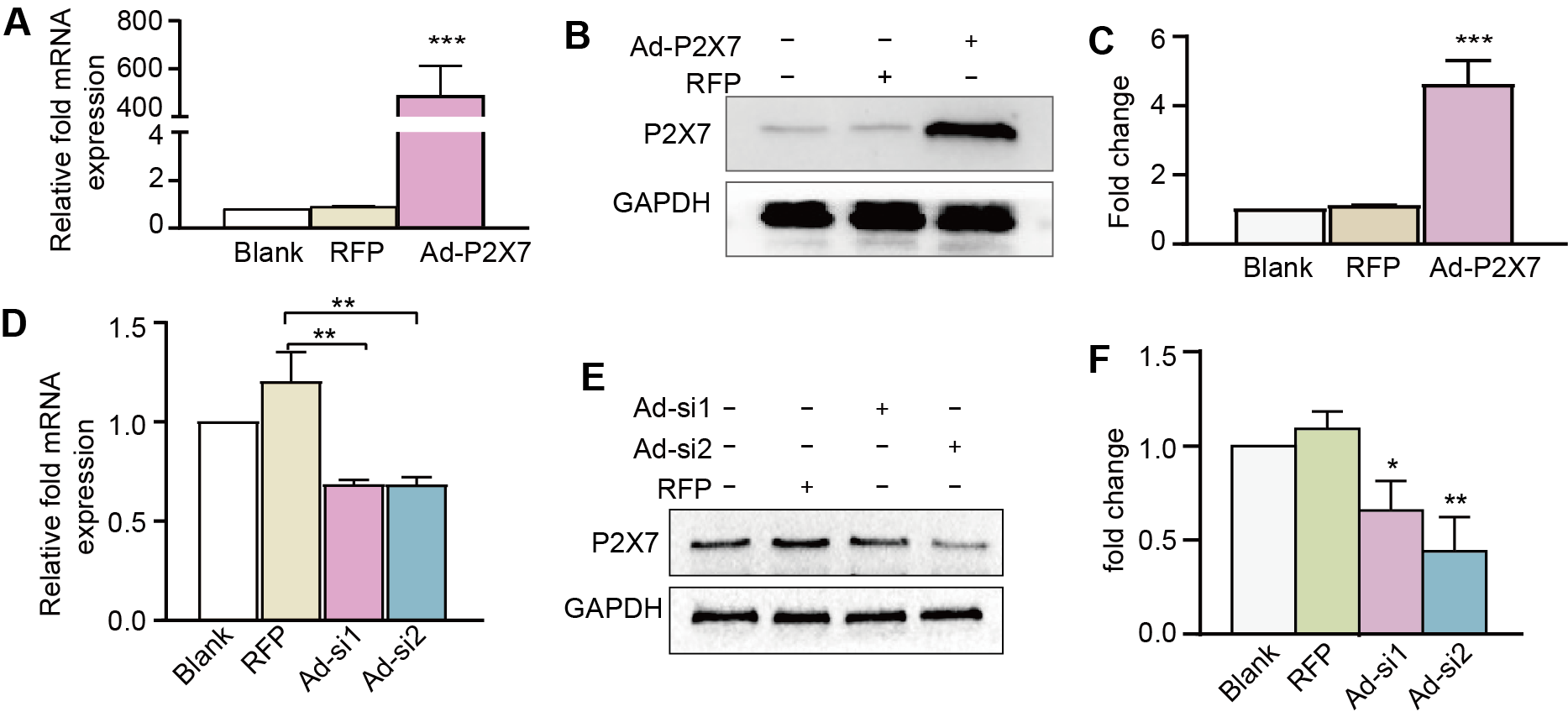


**Supplementary Fig.4 The validation of adenovirus overexpressing P2X7 (Ad-P2X7), and adenovirus expressing small interfering RNA for P2X7 (Ad-si1, and Ad-si2). (A)** qRT-PCR to validate the overexpression of P2X7 by adenovirus Ad-P2X7 in C3H10T1/2 cells. **(B)** Western blot assay to validate the overexpression of P2X7 by adenovirus Ad-P2X7 in C3H10T1/2 cells. **(C)** Quantitative analysis of Western blot results in Supplementary Fig.3B. **(D)** qRT-PCR to validate the silencing of P2X7 by adenovirus Ad-si1 and Ad-si2 in C3H10T1/2 cells. **(E)** Western blot assay to validate the silencing of P2X7 by adenovirus Ad-si1 and Ad-si2 in C3H10T1/2 cells. **(F)** Quantitative analysis of Western blot results in Supplementary Fig.3E. *P < 0.05, **P < 0.01, ***P < 0.001.


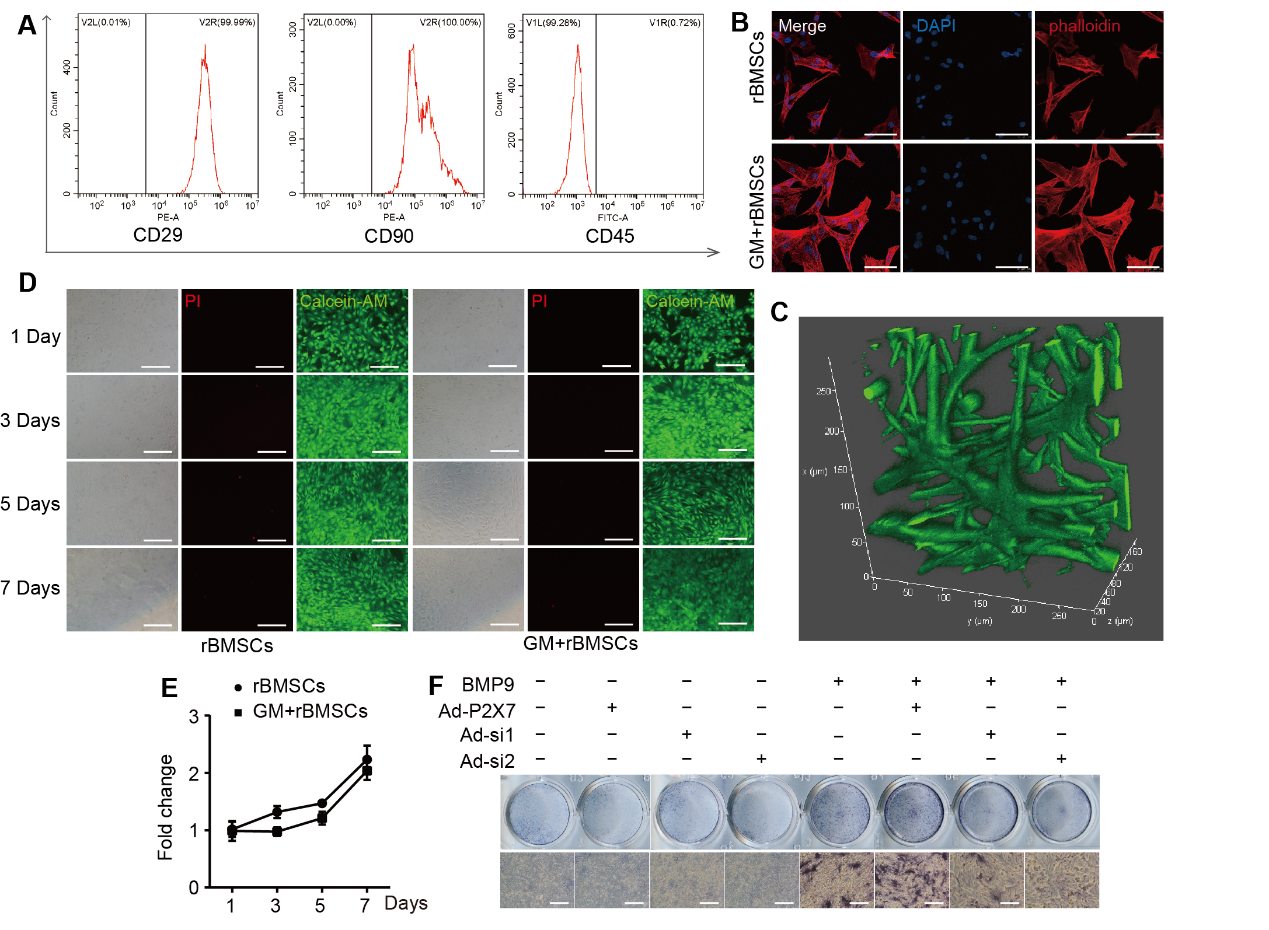


**Supplementary Fig.5 5% GelMA has no significant effect on rBMSCs growth. (A)** Flow cytometry to identify the classical surface markers (CD29, CD90 and CD45) of rBMSCs. **(B)** Confocal microscopy to detect the morphology of rBMSCs treated 5% GelMA. Phalloidin (Red) for cytoskeleton staining; DAPI (Blue) for nuclear visualization. Scale bars, 50 μm. **(C)** Confocal microscopy to visualize the three-dimensional structure of rBMSCs treated with 5% GelMA. **(D)** Live /dead cell double-staining assay to detect the mortality of rBMSCs treated with 5% GelMA. Scale bars, 100 μm. **(E)** MTT assay to assess the cell viability of rBMSCs treated with 5% GelMA. **(F)** ALP staining assay to examine the effect of P2X7 on the osteogenic differentiation of rBMSCs upon BMP9 treatment. Scale bars, 100 μm. *P < 0.05, **P < 0.01, ***P < 0.001.


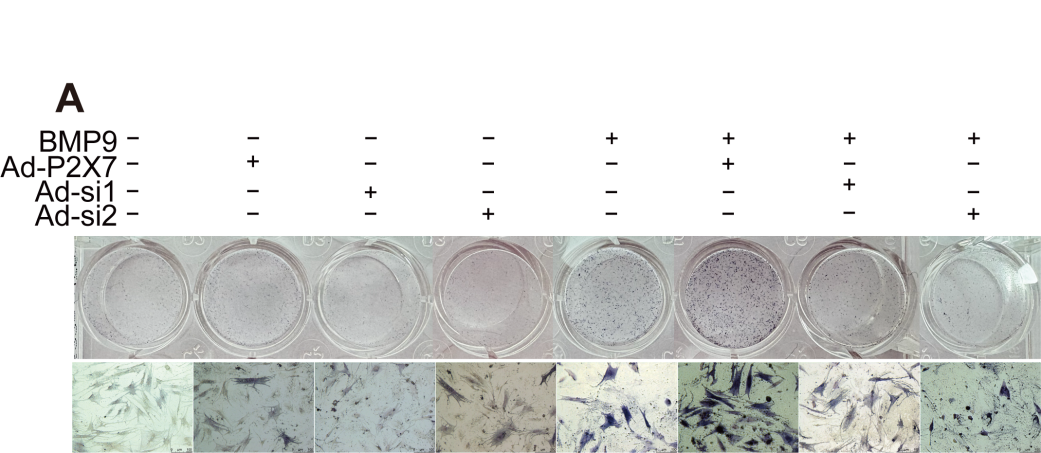


**Supplementary Fig.6** ALP staining assay to examine the effect of P2X7 on the osteogenic differentiation of hBMSCs upon BMP9 treatment. Scale bars, 100 μm.


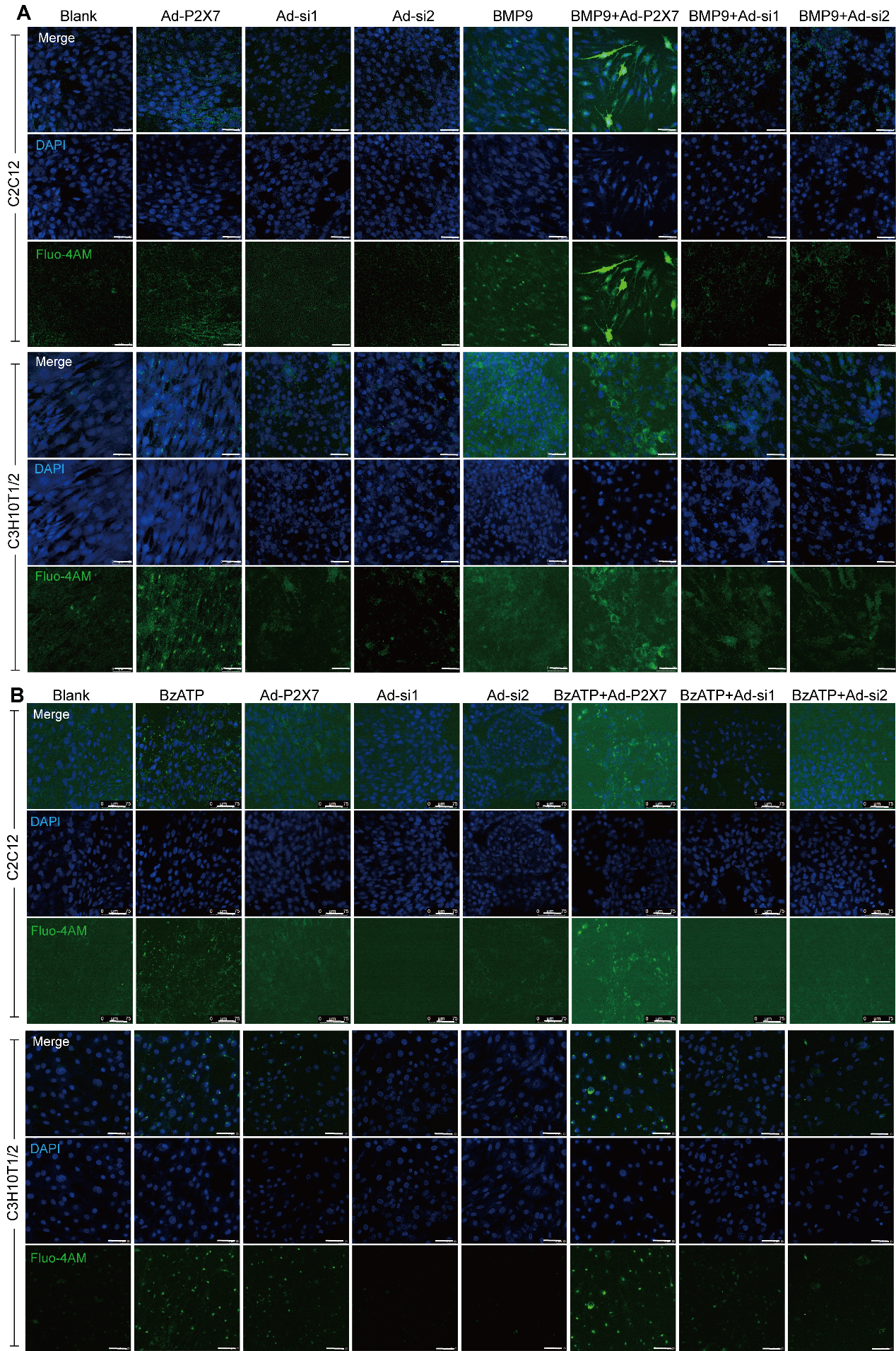


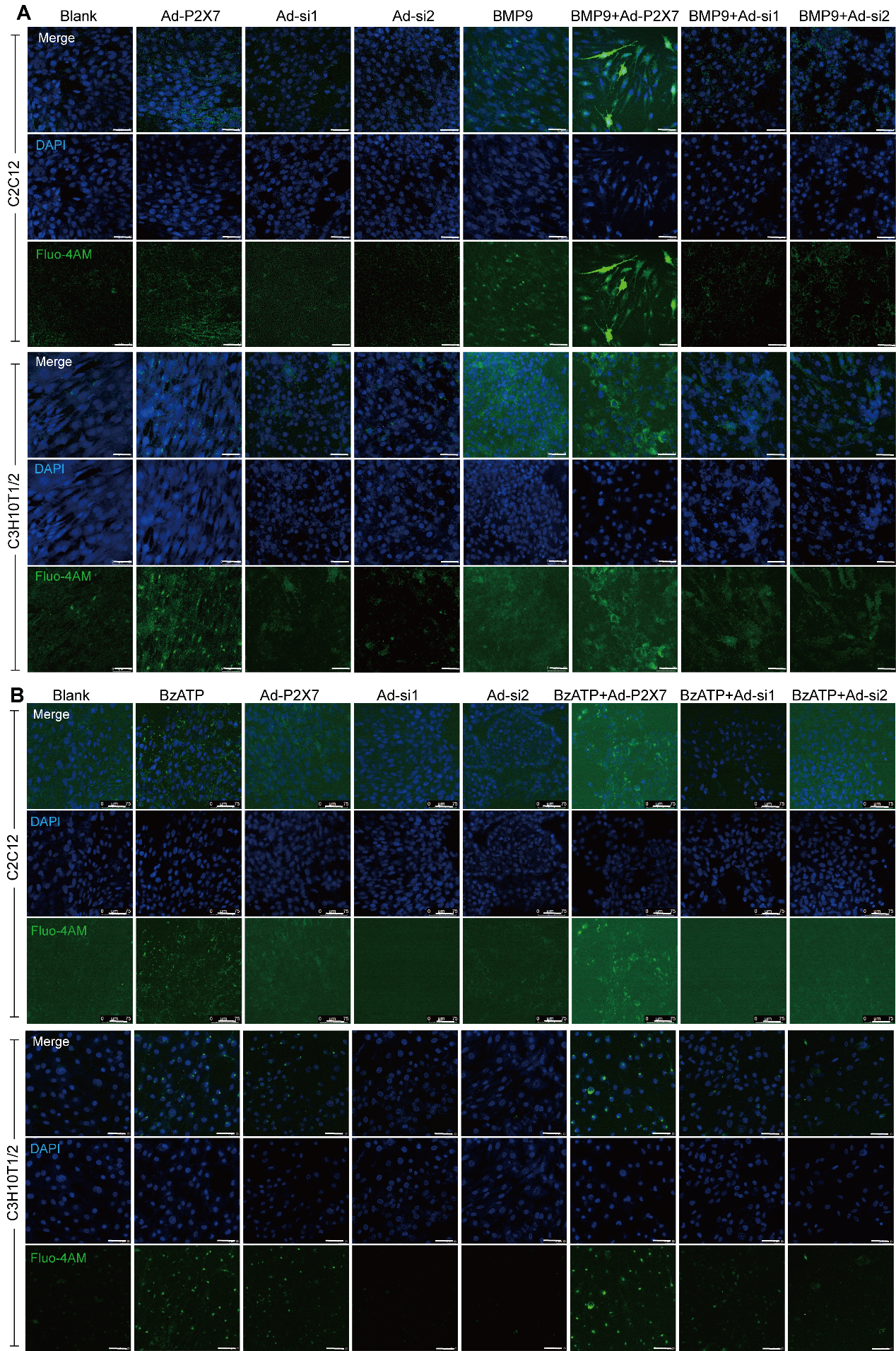


**Supplementary Fig.7 BMP9 promotes intracellular Ca^2+^ accumulation by activating P2X7. (A)** Flou-4AM probe to evaluate the effect of P2X7 overexpression and P2X7 silencing on intracellular Ca^2+^ accumulation of MSCs after BMP9 treatment. Scale bars, 50 μm. **(B)** Flou-4AM probe to evaluate the effect of P2X7 overexpression and P2X7 silencing on intracellular Ca^2+^ accumulation of MSCs after BzATP treatment. Scale bars, 50 μm.

**
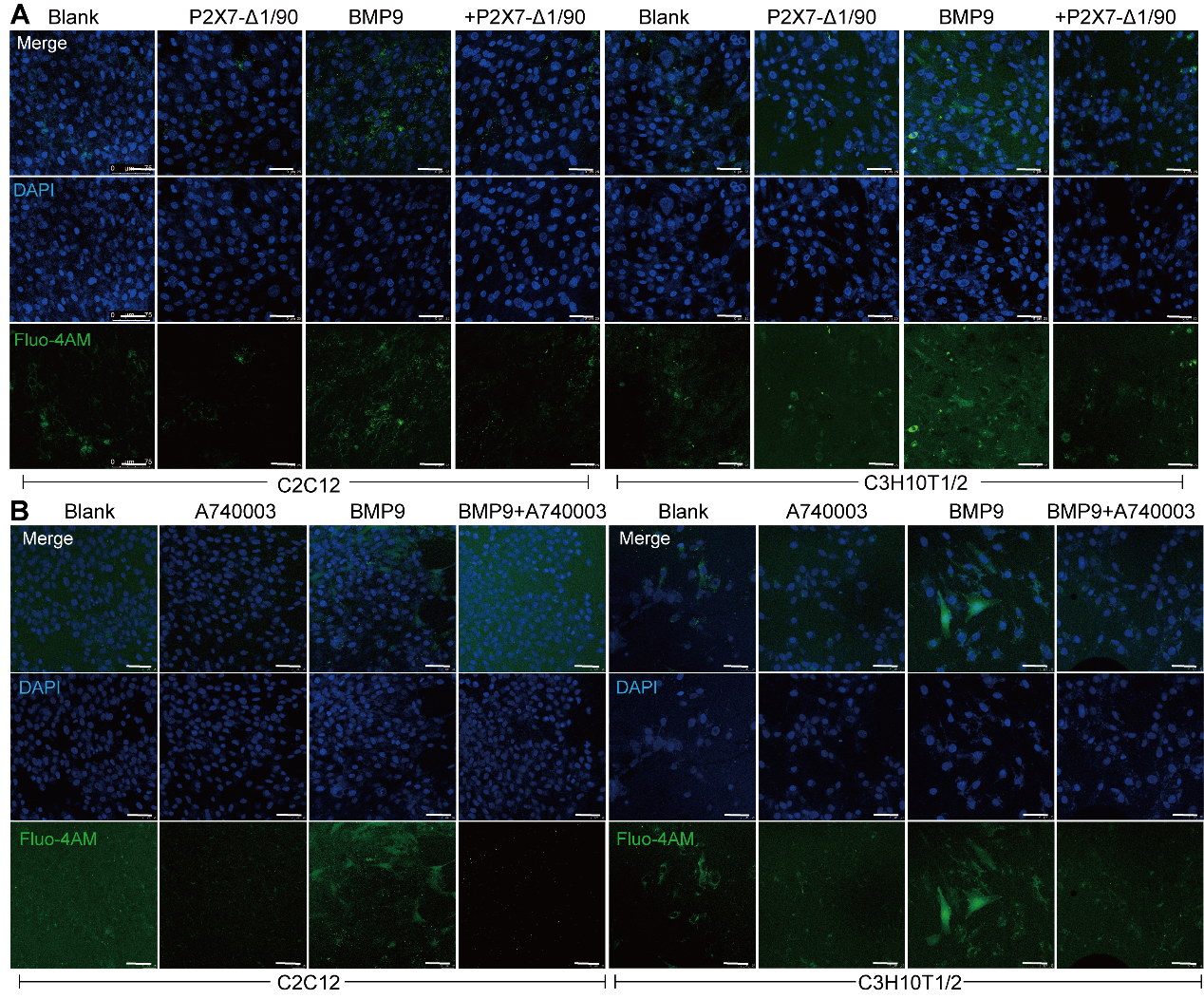
**

**Supplementary Fig.8 BMP9 increases intracellular Ca^2+^ flux by activating P2X7. (A)** Flou-4AM probe to evaluate the effect of P2X7 lost-of-function mutation (P2X7-Δ1/90) on intracellular Ca^2+^ accumulation of MSCs after BMP9 treatment. Scale bars, 50 μm. **(B)** Flou-4AM probe to evaluate the effect of P2X7 inhibitor (A740003) on intracellular Ca^2+^ accumulation of MSCs after BMP9 treatment. Scale bars, 50 μm.

Supplementary Table 1: The primer sequences for qRT-PCR

| Gene | Forward (5'-3') | Reverse (5'-3') |
| --- | --- | --- |
| *P2x1* | GGATGGTGCTGGTACGAAAC | CCACCCAATGACGTAGACCA |
| *P2x2* | AGCATGAGGGTTCACAGCTC | TCCTGTCCGAATCCCATTGC |
| *P2x3* | GAAAAGGCCCCATTTTGCCC | CACACACCCAGCCGATCTTA |
| *P2x4* | TCGTGTGGGAAAAGGGCTAC | GAGCTGGGACCACATAGTCG |
| *P2x5* | GGAGTCTGTTGTAGCGGGAC | TCCGTTGGCATGGACTTTGT |
| *P2x6* | CTCATCCCTACGGCCATCAC | AGTCGTCTTTGGGGCTCTTG |
| *P2x7* | GAGCACGAATTATGGCACCG | TAACAGGCTCTTTCCGCTGG |
| *Bmp9* | TGAGTCCCATCTCCATCCTC | ACCCACCAGACACAAGAAGG |
| *Id1* | ACGACATGAACGGCTGCT | CAGCTGCAGGTCCCTGAT |
| *Id2* | CAGCATCCCCCAGAACAA | TCTGGTGATGCAGGCTGA |
| *Id3* | CTACGAGGCGGTGTGCTG | GCGCGAGTAGCAGTGGTT |
| *Osx* | GGGAGCAGAGTGCCAAGA | TACTCCTGGCGCATAGGG |
| *Runx2* | GGTGAAACTCTTGCCTCGTC | AGTCCCAACTTCCTGTGCT |
| *Gapdh* | GGCTGCCCAGAACATCAT | CGGACACATTGGGGGTAG |

Supplementary Table 2. The detail of primary antibodies.

| Antibody | Manufacturer | Cat. Num | Dilution |
| --- | --- | --- | --- |
| anti-P2X7 | Affinity, China | AF4626 | 1:1000 |
| anti-BMP9 | Affinity, China | DF7758 | 1:1000 |
| anti-Smad4 | Proteintech, China | 10231-1-AP | 1:1000 |
| anti-Smad5 | Proteintech, China | 12167-1-AP | 1:1000 |
| anti-Lamin B1 | Bimake, China | A5106 | 1:1000 |
| anti-RUNX2 | Bimake, China | A5193 | 1:1000 |
| anti-osteocalcin | Affinity, China | DF12303 | 1:1000 |
| anti-osteopontin | Bimake, China | A5427 | 1:1000 |
| anti-phospho-CaMKII beta/gamma/ delta (Thr287) | Affinity, China | AF3434 | 1:1000 |
| anti-CaMKII | Affinity, China | AF6493 | 1:1000 |
| anti-β-catenin | Bimake, China | A5038 | 1:1000 |
| anti-GSK-3β | Affinity, China | AF5016 | 1:1000 |
| anti-p Smad 1/5/9 | Affinity, China | AF8313 | 1:1000 |
| anti-phosphorylated GSK-3β Ser9 | Cell Signaling Technology | 5558 | 1:1000 |
| anti-GAPDH | Affinity, China | AF7021 | 1:2000 |
